# Qualitative network models and genome-wide expression data define carbon/nitrogen-responsive molecular machines in *Arabidopsis*

**DOI:** 10.1186/gb-2007-8-1-r7

**Published:** 2007-01-11

**Authors:** Rodrigo A Gutiérrez, Laurence V Lejay, Alexis Dean, Francesca Chiaromonte, Dennis E Shasha, Gloria M Coruzzi

**Affiliations:** 1 Department of Biology, New York University, Washington Square East, New York, NY 10003, USA; 2 Departamento de Genética Molecular y Microbiología, Pontificia Universidad Católica de Chile. Alameda 340. 8331010. Santiago, Chile; 3 Department of Statistics, Penn State. 326 Thomas Building, University Park, PA 16802, USA; 4 Courant Institute of Mathematical Sciences, New York University. 251 Mercer Street, New York, NY 10012, USA; 5 Biochimie et Physiologie Moleculaire des Plantes, INRA, Place Viala, F-34060 Montpellier Cedex 1, France

## Abstract

**Background:**

Carbon (C) and nitrogen (N) metabolites can regulate gene expression in *Arabidopsis thaliana*. Here, we use multinetwork analysis of microarray data to identify molecular networks regulated by C and N in the *Arabidopsis *root system.

**Results:**

We used the *Arabidopsis *whole genome Affymetrix gene chip to explore global gene expression responses in plants exposed transiently to a matrix of C and N treatments. We used ANOVA analysis to define quantitative models of regulation for all detected genes. Our results suggest that about half of the *Arabidopsis *transcriptome is regulated by C, N or CN interactions. We found ample evidence for interactions between C and N that include genes involved in metabolic pathways, protein degradation and auxin signaling. To provide a global, yet detailed, view of how the cell molecular network is adjusted in response to the CN treatments, we constructed a qualitative multinetwork model of the *Arabidopsis *metabolic and regulatory molecular network, including 6,176 genes, 1,459 metabolites and 230,900 interactions among them. We integrated the quantitative models of CN gene regulation with the wiring diagram in the multinetwork, and identified specific interacting genes in biological modules that respond to C, N or CN treatments.

**Conclusion:**

Our results indicate that CN regulation occurs at multiple levels, including potential post-transcriptional control by microRNAs. The network analysis of our systematic dataset of CN treatments indicates that CN sensing is a mechanism that coordinates the global and coordinated regulation of specific sets of molecular machines in the plant cell.

## Abstract 2

Qualitative network models and genome-wide expression data define carbon/nitrogen-responsive molecular machines in *Arabidopsis *and indicate that regulation by carbon/nitrogen metabolites occurs at multiple levels.

## Background

Integrating carbon (C) and nitrogen (N) metabolism is essential for the growth and development of living organisms. In addition to their essential roles as macronutrients, both C and N metabolites can act as signals that influence many cellular processes through regulation of gene expression in plants [1-6] and other organisms (for example, [7,8]). In plants, C and N metabolites can regulate developmental processes such as flowering time [9] and root architecture [10], as well as several metabolic pathways, including N assimilation and amino acid synthesis (for example, [11,12]). Previous microarray studies from our group and others have identified many genes whose expression changes in response to transient treatments with nitrate [2,13,14], sucrose [5,15] or nitrate plus sucrose [16,17] in *Arabidopsis *seedlings. Addition of nitrate to N-starved plants causes a rapid increase in the expression of genes involved in nitrate uptake and reduction, production of energy and organic acid skeletons, iron transport and sulfate uptake/reduction [2,13]. These changes in gene expression preceded the increase in levels of metabolites such as amino acids, indicating that changes in mRNA levels are biologically relevant for metabolite levels, if a time delay is introduced [13]. Using a nitrate reductase (NR-null) mutant, Wang *et al*. [14] showed that genes that respond directly to nitrate as a signal were involved in metabolic pathways such as glycolysis and gluconeogenesis [14]. Separately, sugars, including glucose and sucrose, have been shown to modulate the expression of genes involved in various aspects of metabolism, signal transduction, metabolite transport and stress responses [5,15].

These studies confirm the existence of a complex CN-responsive gene network in plants, and suggest that the balance between C and N rather than the presence of one metabolite affects global gene expression. However, despite the extensive collection of biological processes regulated by N or C, to date, none of these studies have addressed the possible mechanisms underlying CN sensing, nor the interdependence of the CN responses in a network context. In this study, we use a systematic experimental space of CN treatments to determine how C and N metabolites interact to regulate gene expression. In addition, we provide a global view of how gene networks are modulated in response to CN sensing. For the latter, we created the first qualitative network model of known metabolic and regulatory interactions in plants to analyze the microarray data from a gene network perspective. The combination of quantitative models describing the gene expression changes in response to the C and N inputs and qualitative models of the plant cell gene responses allowed us to globally identify a set of gene subnetworks affected by CN metabolites.

## Results

### A systematic test of CN interactions

**Figure 1:**
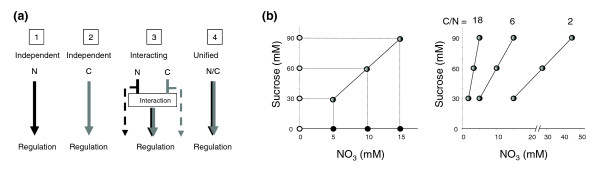
Experimental design to investigate C and N interactions. **(a) **Hypothetical models to explain regulation by C and N metabolites. The four possible models of gene expression response to N and C treatments are illustrated. Model 1 (N independent of C) represents genes that are regulated by N in a manner that is independent of the amount of C present. Model 2 (C independent of N) is equivalent to model 1 but for C. Model 3 represents different types of interactions between C and N. Model 4 represents regulation by the ratio of C/N. In this case, neither C nor N can affect gene expression. Regulation according to all models could be positive or negative, but only positive examples are depicted. **(b) **Systematic experimental space to investigate C and N interactions. To investigate gene responses to C and N, we used experiments where plants were exposed to C, N or C+N. The graphs summarize the experiments carried out. Each point in the graphs corresponds to one experiment. The x-axis indicates the concentration of nitrate used (nitrogen source) in the experiment. The y-axis indicates the concentration of sucrose used (carbon source) in the experiment. For example, points on the x-axis correspond to experiments in which plants were treated with nitrate in the absence of sucrose.

Based on our current understanding of CN regulation, four general mechanisms for the control of gene expression in response to C and N can be proposed: N responses independent of C; C responses independent of N; C and N interactions; or a unified CN response (Figure 1a). To support or reject these modes of control by C and N metabolites, we designed an experimental space that systematically covers a matrix of C and N conditions (Figure 1b). Plants were grown hydroponically in light/dark cycles (8/16 h) for 6 weeks, with 1 mM nitrate as the N source and without exogenous C. They were then transiently treated for 8 h with: 30, 60 or 90 mM of sucrose; 5, 10 or 15 mM nitrate; and nine treatments in which the C/N ratio was kept constant at 2/1, 6/1 or 18/1 with different doses of CN (Figure 1b). Each C/N ratio treatment was represented by 3 different CN treatments, using 30, 60 or 90 mM of sucrose and the corresponding concentrations of nitrate.

We choose to focus on roots of mature plants for several reasons. First, roots have been shown to have a more robust response to nitrogen compared to shoots in *Arabidopsis *[2]. Second, previous global studies of CN treatments focused on gene responses in *Arabidopsis *seedlings, which consist mostly of shoot tissue [5,16]. In contrast, the coordination of C and N sensing and metabolism in the heterotrophic root system (which is a C sink and an N source) is an important response, but the mechanism of control is largely unknown. Finally, the largest proportion of uncharacterized *Arabidopsis *genes is preferentially expressed in roots (RA Gutiérrez, unpublished results), offering the potential to discover new CN-responsive genes.

Gene expression was evaluated using the *Arabidopsis *ATH1 whole genome array from Affymetrix. All experiments were performed in duplicate, with the exception of the 0 mM sucrose/0 mM nitrate experiment, which was performed four times. RNA samples obtained from the roots in each of the 16 treatments were used to hybridize ATH1 chips. Each hybridization was analyzed using Microarray Suite Software version 5.0 (MAS v5.0) software and custom made S-PLUS [18] functions. We used quantitative PCR (Q-PCR) to verify the responses of six selected genes representative of different responses to CN. The 6 genes were tested under 4 different conditions: 0 mM C, 0 mM N; 30 mM C, 0 mM N; 0 mM C, 5 mM N; 30 mM C, 5 mM N. All genes exhibited comparable responses in Q-PCR experiments and microarray data, with a median correlation coefficient when comparing Q-PCR and microarray data of 0.97.

### Hierarchical clustering distinguishes C-, CN- and N-responsive genes in *Arabidopsis *roots

**Figure 2:**
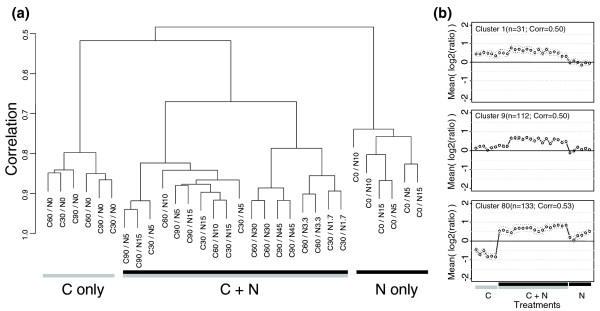
Unsupervised hierarchical clustering analysis suggests various modes of regulation by CN. **(a) **Hierarchical clustering distinguishes three main responses: C alone, N alone and C+N. **(b) **Hierarchical clustering of the gene expression patterns reveals different modes of regulation. Three representative gene expression patterns in response to the CN treatments are shown. The mean expression ± 95% confidence interval of the mean for all genes in the cluster is plotted.

To evaluate the global impact of the different C and N treatments on gene expression in *Arabidopsis *roots, we used unsupervised hierarchical clustering. Figure 2a shows a dendrogram representation of the relationships among the experiments based on these global genome responses. C, N, and CN treatments clustered together and separately from each other, indicating that global genome-wide responses to C, N and CN treatments in roots are distinct. The CN treatment experiments were highly correlated with each other, and clustered together regardless of the CN dose or C/N ratio (Figure 2a).

To analyze the responses of specific gene sets, we carried out a similar cluster analysis on the C-, N-, and CN-responsive genes. Gene clusters with a correlation greater than 0.5 were selected for further examination. Figure 2b shows scatter plots with the average expression of all genes in three representative clusters. Cluster 1 contains 31 genes that had comparable responses in the C and CN treatments, and did not respond to N treatments, suggestive of C-only regulation. Cluster 9 corresponds to 112 genes that were induced only in the CN treatments, suggesting regulation by a CN signal. The 133 genes in cluster 80 were repressed by C, induced by N, and more strongly induced when both C and N were present, suggesting interactions between the responses elicited by C and N metabolites. We found no genome-wide evidence to support the hypothesis that the C/N ratio regulates expression of gene sets under our treatment conditions using either clustering or other statistical methods (data not shown). However, it was clear that N does have a significant interaction with C in regulating genome-wide expression, as many genes were found to respond to N in a C-dependent manner (or vice versa), as exemplified by the genes in cluster 9 and cluster 80 (Figure 2b). In fact, the average expression pattern of many clusters identified showed statistically significant CN interactions as determined by the analysis of variance (AOV p < 0.01), suggesting that model 3 (C and N interactions; Figure 1a) is a prominent mode of regulation in response to C and N treatments in plants.

### A catalogue of molecular responses and interactions between C and N

The clustering analysis above suggested different modes of regulation in response to CN. It also suggested that genome-wide responses to sucrose and nitrate treatments in *Arabidopsis *roots presented three main features: extensive CN interactions; an all-or-nothing response due to the presence of one or both C and N metabolites; and possible CN dose effects. To investigate these hypotheses for the mechanism of CN sensing further, and to classify individual genes based on their response to the treatments, we used AOV to identify the main effects of sucrose and/or nitrate as well as the interaction between these two signals in regulating gene expression. We used regression analysis (LM) to investigate dose dependence. It is important to note that AOV or LM approaches take advantage of all data points simultaneously. As a consequence, our conclusions are more statistically sound than most published microarray results with the Affymetrix platform, which compare two conditions with two to three replicates each.

We found that LM equations did not adequately capture the variability in the data. Determination coefficients (share of explained variability) from the LM fits including individual terms, interaction and second order effects were generally low. In addition, AOV on the residuals of the LM analysis found many genes with significant responses to C, N or CN (data not shown). Instead, we found that AOV analysis was sufficient to explain most of the variability in the data and, consistent with this, LM analysis on the AOV residuals failed to detect any significant coefficient indicative of dose effect. These results suggest that, in the treatments tested, genes followed an 'all-or-nothing' mode of regulation in response to the C and N treatments. Importantly, AOV allowed us to assign quantitative models that characterize the response of each *Arabidopsis *gene to C and N (Table 1). For a graphical representation of the patterns see Figure S1 in Additional data file 2. A complete list of the results can be found in Additional data file 1.

AOV analysis identified 5,341 out of 14,462 detected mRNAs as responding to C and/or N at a 5% false discovery rate. Using this analysis, we found genome-wide support for models 1, 2 and 3 (Figure 1a, Table 1). The largest proportion of genes followed model 2 (C independent of N). By contrast, a comparatively small number of genes responded according to model 1 (N independent of C). The second largest group of genes responded according to variations of model 3 (CN interaction). We found no evidence for model 4 (united or N/C ratio regulation). Consistent with previous findings in *Arabidopsis *seedlings, which consist of mostly shoot tissue [6,16], our analysis suggests that CN or a metabolite product of CN assimilation (for example, an amino acid) may act as a signal to control gene expression in mature *Arabidopsis *roots.

### Interactions between C and N extend beyond metabolism

**Figure 3:**
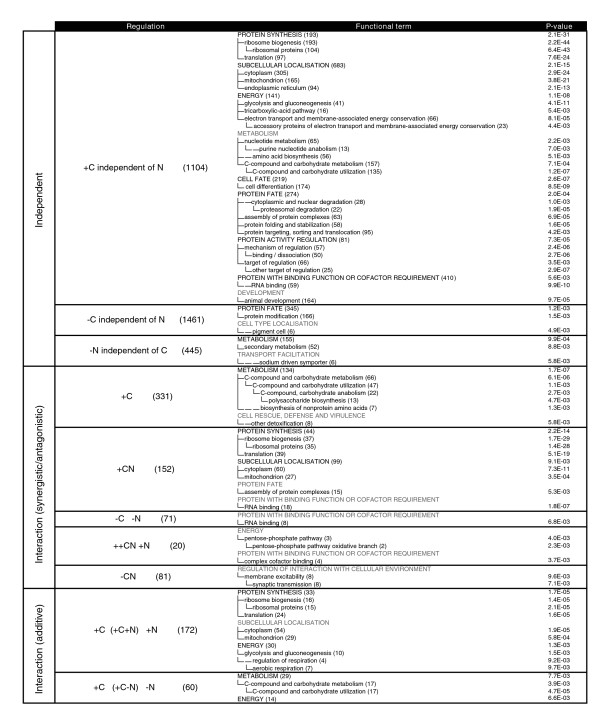
C, N and CN regulation of metabolism and other cellular processes. The number in parenthesis next to each MIPS functional term indicates the number of genes annotated to that term. Categories in gray are not significantly over-represented, but are provided to facilitate data interpretation. The 'Regulation' column shows patterns of regulation as described in Table 1.

To understand the biological significance of the responses to CN treatments, we analyzed the frequency of functional annotations in lists of genes using the BioMaps tool (see Materials and methods). Interestingly, genes regulated by different CN sensing mechanisms (models 1, 2 and 3) showed overlapping functional annotations (Figure 3). That is, the same biological process, for example, protein synthesis, contained genes regulated according to multiple models of CN response. This observation suggests that C and N interact not only at the level of gene expression but also functionally in *Arabidopsis*. Primary and secondary metabolism and energy were predominant biological functions regulated by CN as follows. Genes involved in carbohydrate, nucleotide and amino acid metabolism were induced by C independent of N (model 2). In contrast, N independent of C (model 1) was shown to repress genes involved in secondary metabolism. C and N interacted (model 3) to control the expression of over 200 genes involved in various aspects of primary metabolism, including glycolysis/gluconeogenesis and the pentose-phosphate pathway, among others. In addition to metabolism, other aspects of cellular function, such as protein synthesis, protein degradation, protein targeting and regulation of protein activity, were also over-represented among genes modulated in response to the CN treatments. For example, 193 genes related to protein synthesis and 274 genes involved in protein fate (for example, protein folding, sorting and degradation) were induced by C independent of N (model 2). In addition, 77 other genes related to protein synthesis were induced by a synergistic or additive interaction between C and N (model 3).

### Using a qualitative network model to identify biomodules controlled by C, N and CN interactions

To gain a global, yet detailed, understanding of how the different modes of CN regulation identified above impact molecular processes in the plant cell, we developed a multinetwork tool to integrate information for gene interactions based on a variety of data, including: *Arabidopsis *metabolic pathways; known protein-protein, protein-DNA, and miRNA-RNA interactions; and predicted protein-protein and protein-DNA interactions (described in legend to Figure S2 in Additional data file 2). As a first step towards a molecular wiring diagram of the plant cell, we integrated this information into a multinetwork to generate a qualitative model of the *Arabidopsis *molecular network in which genes are connected by multiple sources of evidence (Figure S2 in Additional data file 2). This *Arabidopsis *multinetwork, which currently has 7,635 nodes and 230,900 edges can be accessed from our accompanying website [19] or through our new VirtualPlant system [20].

**Figure 4:**
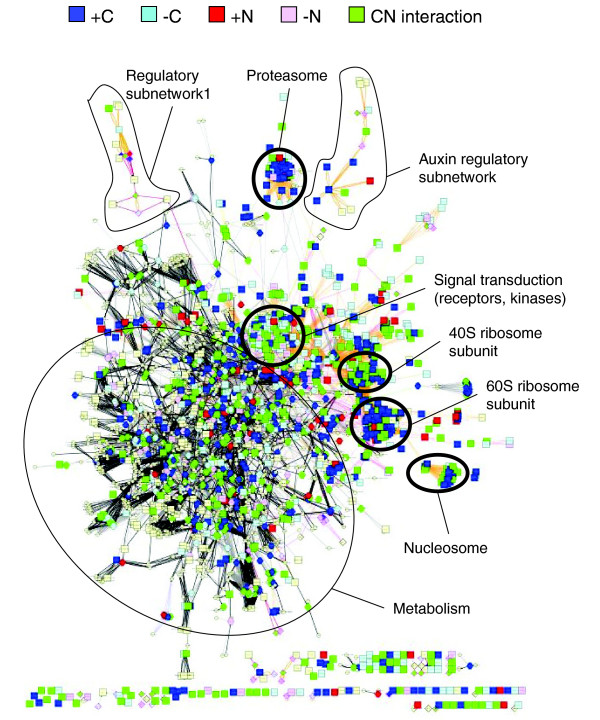
*Arabidopsis *subnetwork controlled by C, N or CN. The different genes and functional associations between them were uniquely labeled and combined into a single network graph. Protein-coding genes, miRNAs, or metabolites are represented as nodes, and color and shapes have been assigned to differentiate them according to function. Edges connecting the nodes represent the different types of biological associations (for example, enzymatic reaction, transport, protein-protein interaction, protein-DNA interaction) and are colored and labeled accordingly. The current version of this *Arabidopsis *multinetwork includes 6,176 *Arabidopsis *genes, 1,459 metabolites (7,635 total nodes) and 230,900 total interactions (edges). We used the open-source Cytoscape software [32] to visualize and query the molecular network for attributes of interest. We used these integrated data as a scaffold on which to analyze the various modes of regulation described above. Because all connections in the network are labeled, the evidence connecting any two nodes or subregions in the network can be readily evaluated. Bold lines represent clusters identified using Antipole (see text for more details). See Figure S3 (in Additional data file 2) for a larger version of this figure.

Figure 4 shows a 'bird's eye' view of the subnetwork generated when we queried the global network described above with the genes from Table 1 that respond to C, N or CN. Visual inspection of the resulting network graph revealed highly connected regions, suggestive of protein complexes or highly connected metabolic or signaling networks (small circles in Figure 4). To address this hypothesis of subnetwork connectivity, we used 'Antipole', a graph clustering algorithm that finds highly connected regions in a network [21]. Some of the clusters identified by Antipole are shown with bold circles in Figure 4. Functional analysis of these clusters (using BioMaps and manual analysis of the gene descriptions) revealed that they corresponded to molecular machines whose expression is coordinated by C and N metabolites. This result indicates that the qualitative network model that we have constructed to summarize and integrate many different data types is a good approximation for the molecular interactions as it is validated by the association of biological components that work together in the plant cell.

Consistent with the functional interaction described above, genes with different models of response to CN were found within the same clusters found by Antipole. For example, many subunits of the 40S and 60S ribosome subunits were induced by C independent of N and, in many instances, also by C in interaction with N. Components of the proteasome were induced by C independent of N, and also by C in interaction with N. Other cellular processes controlled by C, N or CN interactions included chromatin assembly (nucleosome), RNA metabolism, membrane transport, actin cytoskeleton, signal transduction and primary and secondary metabolism. Thus, the network model described above allowed us to identify the metabolic and cellular molecular machines that are interconnected to each other in the larger network and are regulated by C, N or CN interactions.

### CN-responsive regulatory subnetworks

Further analysis of the CN-regulated network enabled us to identify regulatory gene subnetworks that include connected transcription factors and other signaling components. Some of the regulatory genes in the network found to be responsive to the CN treatments include those encoding known regulatory factors crucial for controlling plant growth and development, including: APETALA (At1g68690), CLAVATA1 (At3g49670), as well as several scarecrow-like transcription factors. The CN-regulated network also included teosinte-branched, cycloidea, PCNA factor (TCP) transcription factors repressed by C independent of N (At3g47620, At1g58100), N-independent of C (At4g18390) and CN interactions (At1g53230) as well as one induced by C independent of N (At2g30410). Therefore, and as previously proposed [22], part of the coordinated response of the network of ribosomal genes observed in our CN treatments could potentially be mediated by these associated TCP transcription factors in the gene network. Overall, we found 299 known or putative transcription factors in the network that are regulated by C, N or CN. These genes likely represent only a subset of the regulatory capacity observed to be responsive to the CN treatments in this network. For example, we found a highly connected subdomain of the network involved in signal transduction, including putative receptors of unknown function, protein kinases and protein phosphatases. In addition, we found 27 genes regulated in our experiments, and included in the network, that are known targets of miRNAs. This result suggests that miRNAs may play a role in post-transcriptional regulation of gene expression in gene networks that respond to CN metabolite signals in plants.

The network analysis also highlighted the role of plant hormones in adjusting plant physiology to different CN regimes. We found several regulatory subnetworks in the CN network, in which factors involved in hormone responses are connected by multiple edges, including protein-protein or protein-DNA interactions. One such subnetwork appears to be involved in responses to auxin, as it contains 13 genes in the auxin response pathway: 8 encoding indoleacetic acid-induced proteins (IAAs; At4g14560, At1g04550, At2g33310, At1g51950, At3g23030, At1g04240, At2g22670, At1g04250); 3 encoding auxin-responsive factors (ARFs; At5g62000, At1g59750, At1g19850); the auxin receptor *TIR1 *(At3g62980); and *ASK1 *(At1g10940). In addition, 5 auxin efflux carriers (At1g76520, At2g17500, At5g01990, At1g73590, At1g23080) and 2 auxin transport proteins (At5g57090, At2g01420) were found regulated in our experiments, mostly repressed by N or CN (Table 2).

**Figure 5:**
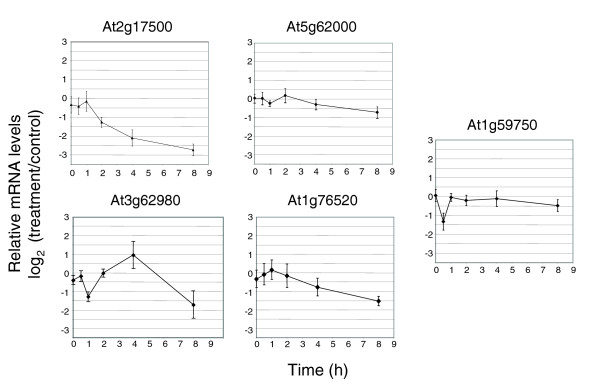
Time course of CN response for genes involved in the auxin response. We monitored the mRNA levels over time for five genes selected from Table 2. We performed three biological replicates, each with a technical replicate. Each graph shows the average expression and standard error of the mean for at least five data points. All mRNA levels were normalized to clathrin. Y-axis, average log2 (treatment/control); x-axis, time in hours. At2g17500, auxin efflux carrier family protein; At1g59750, auxin-responsive factor (ARF1); At1g76520, auxin efflux carrier family protein; At5g62000, transcriptional factor B3 family protein/auxin-responsive factor; At3g62980, transport inhibitor response 1 (TIR1).

To verify the role of these genes in the CN response, we performed time course analysis after C+N addition. Two week old *Arabidopsis *plants grown hydroponically were exposed to treatment (5 mM KNO_3 _+ 30 mM sucrose) or control (5 mM KCl + 30 mM mannitol) conditions for 0.5, 1, 2, 4 and 8 h. We used Q-PCR to monitor the mRNA levels of *TIR1*, two auxin-response factors and two auxin efflux carriers. The Q-PCR data at the 8 h time point were comparable to those obtained by microarrays (Figure S4 in Additional data file 2). As shown in Figure 5, the two auxin-response factors showed similar response patterns, with a modest decrease by 8 h. Both auxin efflux carriers were repressed by the C+N treatments, with the lowest level of expression observed at 8 h. *TIR1 *mRNA levels were also significantly repressed by C+N treatment at 8 h. *TIR1 *mRNA levels appeared to increase by 4 h, but *t*-test failed to detect a significant induction at this time point (0.05 significance). These results confirm that the auxin pathway is modulated by CN metabolites and suggest that the phytohormone auxin acts as a regulator of plant growth in response to C and/or N availability.

## Discussion

In this study, we systematically address the interactions of C and N signals in regulating gene networks by testing the effect that the C background has on global N responses, and vice versa. We tested a systematic experimental space of CN treatments that allowed us to model a quantitative mechanism by which C and N metabolites interact to regulate gene expression in *Arabidopsis *roots. The combination of quantitative models describing the gene expression adjustments in response to C and N inputs, with the analysis of microarray data to generate qualitative models of plant gene networks, allowed us to identify interconnected biomodules of metabolic and cellular processes that are responsive to C and/or N signals.

We used unsupervised clustering to explore the nature of the CN responses in *Arabidopsis *roots. This analysis provided the guidelines that were used for a more rigorous statistical analysis. We found that AOV analysis was sufficient to explain most of the variability in the expression data, and allowed us to assign quantitative models that characterize the response of each *Arabidopsis *gene to C and N. Importantly, many genes previously identified as N or C responsive were found to be regulated by some type of CN interaction in our study (model 3). For example, a previous study identified 1,176 genes regulated in *Arabidopsis *roots in response to a 20 min NO_3_^- ^treatment [2]. Out of the 1,176 genes from that previous nitrate study, 667 had reliable responses in our dataset, and were assigned to a CN-regulatory model class as described in the previous section. Of these 667 genes, we found 149 genes (22%) to be exclusively N responsive in our treatment conditions. By contrast, our study shows that 78% of the nitrate inducible genes were in fact regulated by N interactions with C. These genes include those encoding enzymes and transporters associated with N assimilation functions, such as nitrate transport and nitrate reduction. Therefore, a large proportion of previously reported N-responsive genes may exhibit modulation depending on the carbon background. Similarly, we were able to assign a regulatory pattern for 523 genes of the 978 genes that were previously reported to be regulated by C [17]. Of these 523 C-regulated genes, only 91 (17%) followed a 'C independent of N' mode of regulation in our treatment conditions (model 2 in Figure 1a). Thus, our data show for the first time that a large portion of the previously reported C-responsive genes (83%) may in fact respond to C in interaction with N. In contrast, only 6 out the 2,565 genes found in our study to follow model 2 in our classification method (C independent of N), were reported to be regulated by CN in previous studies [13,14,17].

Our results indicate a major role for CN interactions, which is a more prominent regulatory mechanism than previously suggested. In addition, they suggest that systematic experimental designs that cover a large range of treatment conditions not only allow one to infer quantitative models of gene responses, but are also more effective at detecting gene regulation than traditional approaches with only one treatment and control. Overall, a combined total of 9,417 genes were found to respond to C, N or CN in our study or at least one other published experiment. This indicates that a much greater portion of the *Arabidopsis *transcriptome is controlled by C and/or N metabolites than previously thought.

Previous studies on individual genes suggested that the C/N ratio may be an important signal for the control of gene expression in plants [23]. The systematic experimental space used in our study allowed us to evaluate the significance of C/N ratio differences for the control of global gene expression in *Arabidopsis *roots. For a gene to be regulated by the C/N ratio, similar gene expression levels are expected whenever the ratio is the same, regardless of the dose of the nutrient signals. Similarly, ratio-responsive genes would be expected to exhibit different responses when the ratio is altered. We compared the mRNA levels of genes at C/N ratios of 2/1, 6/1 and 18/1. Clustering, ANOVA and correlation analysis failed to detect any significant ratio-dependent control of global gene expression in our conditions (data not shown). This result suggests that the C/N ratio model (model 4 in Figure 1) is likely not a major regulatory mechanism, at least under the conditions tested. Instead, our results are consistent with the hypothesis that the ratio or balance between C and N is sensed through C- and N-responsive pathways that intersect at either the signaling level or the metabolite level (for example, a CN metabolite).

The interdependence of C and N is most evident when analyzing the putative functions of genes regulated by C and/or N metabolites. The genes we identified as regulated by models 1 (C independent of N), 2 (N independent of C) and 3 (CN interaction) showed functional overlap with regard to control of biological processes. This means that a single biological process contained genes regulated according to different models of C and/or N response. Primary and secondary metabolism are predominant functions that exhibited modulation by C and/or N. In addition to metabolic functions, categories related to various aspects of protein metabolism, including protein synthesis, degradation, targeting and regulation of protein activity, are also over-represented among genes modulated in response to the C and/or N treatments. These results suggest that C and N signals are required to coordinate the synthesis of cytoplasmic and organellar proteins in *Arabidopsis *roots, and that protein synthesis is highly sensitive to the CN status of the plant.

The large number of genes found to be regulated by C and/or N in this study constituted a technical challenge for placing the results in a biological context. The first logical step to address the molecular mechanisms underlying the biological associations of genes is to analyze their properties in the context of what is known. However, this task was impractical considering that we had to analyze several thousand genes. We found that integrating existing knowledge into a relatively simple qualitative network graph greatly simplified the task of extracting biological meaning from the microarray data and finding functional associations between CN regulated genes. Using the genes regulated by C, N or CN as a query, we were able to identify a gene subnetwork of 2,620 interconnected genes that is modulated by these metabolite treatments. Visual inspection of the resulting gene network graph revealed highly connected subregions, suggestive of protein complexes or highly connected metabolic or signaling networks. Further graph clustering analysis and functional annotation of the resulting clusters confirmed the biological identity of these subnetworks as biological modules or molecular machines controlled by C and/or N. For example, protein synthesis and protein degradation machineries are regulated by the C or CN treatments. Other processes represented in CN regulated biomodules include chromatin assembly (nucleosome), RNA metabolism, transport, actin cytoskeleton formation, signal transduction and many aspects of metabolism.

We found that C and/or N could regulate gene expression at multiple levels. We found known or putative transcription factors to be regulated in our CN treatments. However, transcriptional control is likely to represent a subset of the mechanisms involved in adjusting gene product levels in response to various CN regimes. We found many signal transduction components in the CN gene network, including genes of unknown function that are likely to code for putative receptors, protein kinases and protein phosphatases in this CN network. Interestingly, we also found that the CN gene network contained many components of the ubiquitin-mediated protein degradation pathway controlled by C, N or by CN interaction. In addition, we found known targets of miRNAs to be CN regulated in the gene network. These results suggest that post-transcriptional control by miRNAs and protein degradation play a prominent role in the regulation of gene expression and controlling gene product levels in response to CN metabolites in plants.

The potential role of auxin in adjusting plant physiology to different CN regimes was also evident from the multinetwork analysis. Interestingly, the *Transport Inhibitor Response 1 *(*TIR1*) gene expression was regulated by both C and N. *TIR1 *is thought to encode the auxin receptor [24]. This regulation of expression of the auxin receptor could provide a point of integration for C and N responses in *Arabidopsis*. Auxin has been proposed as a systemic signal involved in shoot to root communication of the N status of the shoot [25]. In addition to regulatory factors known to act in the auxin signaling pathway (ARF and IAA proteins), we found genes coding for auxin efflux carriers and auxin transport proteins in the gene network, suggesting that auxin transport in the root may be directly regulated by N and C. This supports a model in which N regulation of auxin transport and auxin responses in the root may allow the root to adjust growth and development as a function of the local N supply.

The results of our CN network analysis provide a starting point for future studies by identifying the regulatory factors - or network hubs - that are likely to be important for the regulation of gene networks in *Arabidopsis *roots in response to CN. By combining existing knowledge into qualitative network models, and using this as a scaffold on which to interpret microarray data, this allowed us to identify molecular machines controlled by C and/or N. As more genome-wide information about plant gene interactions becomes available, the predictive power of such multinetwork models will increase. We hope that this work on CN regulatory gene networks serves as an exemplar for the integration and analysis of genome wide datasets in *Arabidopsis*, and that our qualitative network model described herein will become a valuable resource for the scientific community.

## Materials and methods

### Plant growth and treatments

We used *Arabidopsis thaliana *Col-0 for all experiments. The plants were grown hydroponically on nutrient solution as described previously [26]. Briefly, plants were grown on sand, placed in custom-designed styrofoam rafts in a growth chamber (EGC, Chagrin Falls, OH, USA) at 22°C with 60 mmol photons m^-2^s^-1 ^light intensity and 8 h/16 h light/dark cycles. The seeds were initially germinated in tap water. After one week, the water was replaced with a complete nutrient solution [26]. All the experiments were performed with six week old plants. Nutrient solutions were renewed weekly and on the day before the experiments. For treatments, individual rafts were transferred to containers with 300 ml of nutrient solution supplemented with various concentrations of nitrate (as a mix of 2/1 KNO_3_/Ca(NO_3_)_2_) and/or sucrose. The N-free nutrient solutions contained 0.25 mM K_2_SO_4 _and 0.25 mM CaCl_2 _instead of KNO_3 _and Ca(NO_3_)_2_. Plants were transferred to treatment media at the beginning of the light period and were harvested 8 h afterwards. Roots and leaves were harvested separately and quickly frozen in liquid N_2_. All experiments were carried out in duplicate with the exception of the no sucrose/no nitrate treatment, which was performed four times. For the time course experiments, plants were grown hydroponically in phytatray boxes (P1552, Sigma, St. Louis, MO, USA) with Murashige and Skoog basal medium (Formula 97-5068EA, GIBCO, Grand Island, NY, USA) supplemented with 3 mM sucrose and 1 mM NH_4 _as ammonium succinate (205049, MP Biomedicals, LLC. Solon, OH, USA). Plants were transferred to treatment media at the beginning of the light period and harvested 0.5, 1, 2, 4, or 8 h afterwards. Roots were harvested and quickly frozen in liquid N_2 _for RNA isolation.

### Microarray hybridization

Total RNA extraction was performed as described previously [27]. cDNA was synthesized from 8 μg total RNA using T7-Oligo(dT) promoter primer and reagents recommended by Affymetrix (Santa Clara, CA, USA). Biotin-labeled cRNA was synthesized using the Enzo BioArray HighYield RNA Transcript Labeling Kit (Enzo, New York, NY, USA). The concentration and quality of the cRNA was evaluated by A_260_/_280 _nm reading and 1% agarose gel electrophoresis. We used 15 μg of labeled cRNA to hybridize the *Arabidopsis *ATH1 Affymetrix gene chip for 16 h at 42°C. Washing, staining and scanning were performed as recommended by Affymetrix. Image analysis and normalization to a target median intensity of 150 was performed with the Affymetrix MAS v5.0 set at default values. We analyzed the reproducibility of replicates using the correlation coefficient and visual inspection of scatter plots of pairs of replicates. We discarded one of the four replicates for the 0 C/0 N treatment because of poor reproducibility. All raw and normalized data are available from the ArrayExpress database [28] under experiment E-MEXP-828.

### Clustering analysis

For clustering analysis all the individual treatments were compared against the three replicates of the 0 C/0 N treatment. The three comparisons were processed as follows. First, all data points with absent calls (MAS v5.0 quality control) in both treatment and baseline hybridizations were labeled with 'NA' values. Second, data points with an absent call in one hybridization and present call in the other hybridization were required to have a raw intensity of ≥100. Third, data points in which two or more replicates were not consistent (different change calls by MAS v5.0) were labeled with 'NA'. All ratios were expressed as log_2_(treatment/control). These processed files were used for hierarchical clustering using the S-PLUS hclust() function with the average linkage method and correlation as similarity metric. Clusters were defined with the cutree() function at a 0.5 correlation cutoff.

### Analysis of variance and regression analysis of expression patterns

AOV and regression analysis (LM) were carried out using the S-PLUS aov() and lm() functions, respectively. The AOV equation used was:

*Y *= μ + *α*_*sucrose *_+ *α*_*nitrate *_+ *α*_sucrose*nitrate _+ ε

where *Y *is the response (expression of a gene represented by the normalized signal reported by the MAS v5.0 software.), μ is the global mean and the alpha coefficients correspond to the effects of sucrose, nitrate and the interaction between sucrose and nitrate, respectively. The LM equation used was:

*Y *= *b*_0 _+ *b*_1_*sucrose + *b*_2_*nitrate + *b*_3_*(sucrose*nitrate) + ε

where *Y *is the response and the *b*_0 _to *b*_3 _coefficients correspond to the intercept, the dose effects of sucrose and nitrate and their interaction, respectively. We tested additional equations for the LM analysis but found no significant improvement in *r*^2 ^values. We used the z scores of the concentrations as predictors in the LM. Each gene was analyzed separately with the AOV and LM equations. Then, the residuals from AOV were subjected to LM analysis and the residuals from LM analysis were subjected to AOV analysis. The equations were the same, but with *Y *replaced by the residuals. We addressed multiple testing by controlling the false discovery rate at 5% as described previously [29]. Patterns of regulation as shown in Table 1 were defined based on the coefficients that were found to be significant by the AOV analysis. Whenever the interaction term was significant, contrasts were used to assess the contribution of the main effects. The 95% confidence interval of the mean in the C, N and/or CN treatments was used to rank the effects when two or more coefficients were found to be significant. All analysis was carried out in S-PLUS using existing or custom made functions.

### Gene-expression profiles using quantitative PCR

Q-PCR was performed as before [30]. Briefly, 1 μg of total RNA was used for cDNA synthesis using the Thermoscript RT-PCR kit (Invitrogen Life Technologies, Carlsbad, CA, USA). Reverse transcription was performed with 1 μg of total RNA and oligo(dT)_20 _as a primer. cDNAs were used for real time Q-PCR with the LightCycler instrument (Roche Diagnostics, Mannheim, Germany). Each mRNA value was corrected by the measurements obtained in the same sample for clathrin (At4g24550) mRNA. The primer sequences utilized were: At1g59750 (forward, AACTTGAGCCCCTAGT; reverse, CTACAGCGACAGCACC), At2g17500 (forward, TTACGTTCTTCGGCAGT; reverse, GTGAGGGCCAGTATCG), At1g76520 (forward, ATGCGTGTGCTATCGA; reverse, GCTTCCGTGCCGATTA), At5g62000 (forward, CAAGCTCAGGCTAGGG; reverse, CCAGCTCAGCGACTAA), At3g62980 (forward, CTCGCGTAGGTCCTTG; reverse, CACTGGTGGGTACACT), At4g24550 (forward, ATACACTGCGTGCAAAG; reverse, TTCGCCTGTGTCACAT). We used SYBRG for all genes, except clathrin, with the Light Cycler DNA Master SYBR Green I kit (Roche Diagnostics). For clathrin the following probes were used: AAGAAGCAGGGCCAGT--FL, LC Red640-GCATGACGTTCACGATACCTATGT--PH with the Light Cycler DNA Master Hybridization Probes kit (Roche Diagnostics).

### Functional analysis in lists of genes

Functional analysis was performed using the classification scheme developed by the Munich Information Center for Protein Sequences (MIPS) [31]. The frequency of each individual MIPS functional term in a list of genes was compared to the frequency of the term in the whole genome. A *p *value of over-representation was then calculated using the hypergeometric distribution. To correct for multiple testing we used a clique approach: we multiplied the unadjusted *p *values by the minimal number of nodes from which all other nodes can be inferred. Terms that are found statistically over-represented are displayed in a color-coded network graph using the View package from GO-TermFinder module. An interface to the program used to perform this analysis, BioMaps, is available on the web [20]. BioMaps is a modification of the GOTerm-Finder package developed by Gavin Sherlock and available from CPAN.

## Additional data files

The following additional data are available with the online version of this paper. Additional data file 1 is a complete list of gene expression patterns and gene annotation. This file contains detailed information about the regulation and annotation for the genes. This file supports Table 1 in the main text. Additional data file contains graphical representations of the different gene expression responses and network views. This file contains four supplemental figures. Figure S1: centroid plots for each pattern determined by AOV as indicated in the Results section. Figure S2: bird's-eye view of the gene network model. Figure S3: higher resolution version of Figure 4. Figure S4: comparison of microarray and Q-PCR data. Additional data file 3 contains all interaction information collected to produce the network model used in this paper. Additional data file 4 contains the legend for each edge label used in Additional data file 3. Additional data file 5 contains the CHP files generated with the Affymetrix MAS v5.0 software as described in the Materials and methods.

## Supplementary Material

Additional data file 1 Detailed information about the regulation and annotation for the genes. This file supports Table 1 in the main text. Click here for file

Additional data file 2 Four supplemental figures. Figure S1: centroid plots for each pattern determined by AOV as indicated in the Results section. Figure S2: Bird's-eye view of the gene network model. Figure S3: higher resolution version of Figure 4. Figure S4: Comparison of microarray and Q-PCR data. Click here for file

Additional data file 3 All interaction information collected to produce the network model used in this paper. Click here for file

Additional data file 4 The legend for each edge label used in Additional data file 3. Click here for file

Additional data file 5 CHP files generated with the Affymetrix MAS v5.0 software as described in the Materials and methods. Click here for file

## Figures and Tables

**Table 1:** Different modes of regulation in response to CN

Mode of regulation	Number of genes	Model
No response	9,121	NA
-N independent	445	1
+N independent	319	1
-C independent	1,461	2
+C independent	1,104	2
+C	331	3
-C	157	3
+CN	152	3
-C -CN -N	103	3
-CN	81	3
+N	76	3
-C -N	71	3
-N	49	3
--C -CN	40	3
+C +N	33	3
++C +CN	28	3
++CN +N	20	3
-CN +N	17	3
+C -CN	16	3
++C +CN +N	15	3
-C +CN	9	3
-C -CN +N	5	3
--CN -N	2	3
+C -CN -N	2	3
+CN -N	1	3
-C +CN +N	1	3
Int	337	3
+C (+C-N) -N	60	3 (additive)
-C (-C-N) -N	136	3 (additive)
+C (+C+N) +N	172	3 (additive)
-C (-C+N) +N	98	3 (additive)

Combinations of letters and plus or minus signs denote the effect of the inputs on regulation of gene expression (for example, +C indicates induction in treatments with carbon). The number of plus or minus signs indicates relative strength of induction (or repression). For model 3, response is observed only for those conditions indicated. For example, +C in model 3 indicates induction in treatments with carbon only and no response for C+N or N treatments. The last four rows of the table contain patterns of additive interactions between C and N. For these patterns of regulation, expression of genes in the C+N treatments was equivalent to adding the expression level in the C-only and the N-only treatments. For a graphical representation of the patterns see Figure S1 (in Additional data file 2). Int, interaction term was found significant by ANOVA analysis but small differences in gene expression between treatments precluded classification by *post hoc *analysis. This group was not analyzed further.

**Table 2:** Auxin regulatory subnetwork

Pattern	PUB_LOCUS	TIGR annotation
-N independent	At2g17500	Auxin efflux carrier family protein
-N independent	At5g01990	Auxin efflux carrier family protein
-N independent	At1g23080	Auxin efflux carrier protein
-N independent	At2g01420	Auxin transport protein
-N independent	At1g59750	Auxin-responsive factor (ARF1)
-N independent	At1g10940	Serine/threonine protein kinase, similar to serine/threonine-protein kinase ASK1
-N independent	At1g19850	Transcription factor MONOPTEROS (MP)/auxin-responsive protein (IAA24)/auxin response factor 5 (ARF5).
-C (-C-N) -N	At1g76520	Auxin efflux carrier family protein
-C (-C-N) -N	At5g62000	Transcriptional factor B3 family protein/auxin-responsive factor.
-C independent	At2g33310	Auxin-responsive protein/indoleacetic acid-induced protein 13 (IAA13)
-C -CN -N	At1g51950	Auxin-responsive protein/indoleacetic acid-induced protein 18 (IAA18)
-C -N	At1g04550	Auxin-responsive protein/indoleacetic acid-induced protein 12 (IAA12)
-CN +N	At3g62980	Transport inhibitor response 1 (TIR1) (FBL1) E3 ubiquitin ligase SCF complex F-box subunit
+N independent	At3g23030	Auxin-responsive protein/indoleacetic acid-induced protein 2 (IAA2)
+C independent	At1g73590	Auxin efflux carrier protein, putative (PIN1) identical to putative auxin efflux carrier protein; AtPIN1
+C independent	At5g57090	Auxin transport protein (EIR1)
+C independent	At4g14560	Auxin-responsive protein/indoleacetic acid-induced protein 1 (IAA1)
+C independent	At1g04250	Auxin-responsive protein/indoleacetic acid-induced protein 17 (IAA17)
+C independent	At1g04240	Auxin-responsive protein/indoleacetic acid-induced protein 3 (IAA3)
+C independent	At2g22670	Auxin-responsive protein/indoleacetic acid-induced protein 8 (IAA8)
