# Supplementary material for: Qualitative network models and genome-wide expression data define carbon/nitrogen-responsive molecular machines in Arabidopsis
Source: Genome Biol. 2007 Jan 11;8(1):R7. doi: 10.1186/gb-2007-8-1-r7 (PMC1839130; doi:10.1186/gb-2007-8-1-r7)
Supplement: Additional data file 2 — Four supplemental figures. Figure S1: centroid plots for each pattern determined by AOV as indicated in the Results section. Figure S2: Bird's-eye view of the gene network model. Figure S3: higher resolution version of Figure 4. Figure S4: Comparison of microarray and Q-PCR data. [file gb-2007-8-1-r7-S2.ppt]

## Slide 1
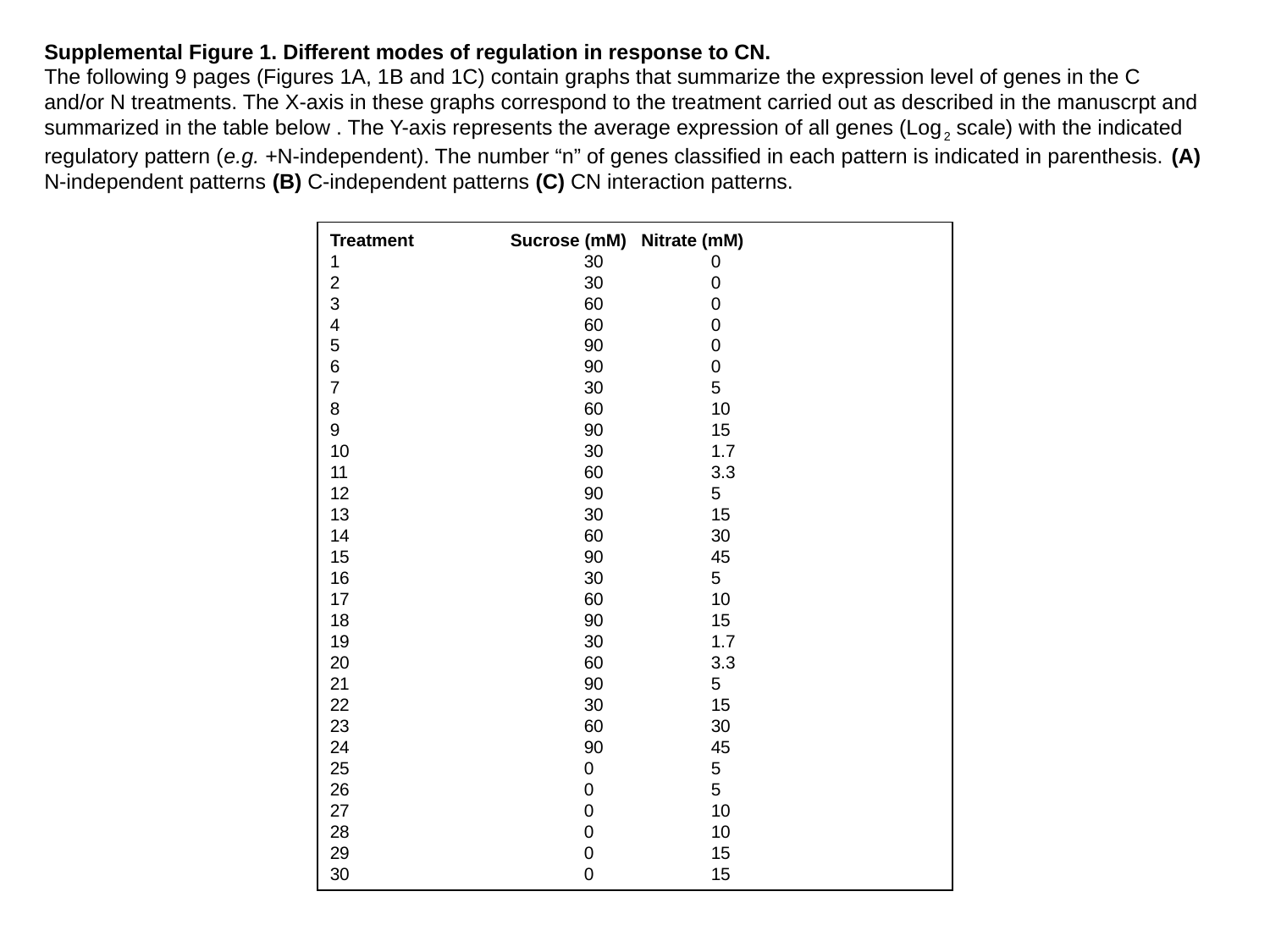

Supplemental Figure 1. Different modes of regulation in response to CN.
The following 9 pages (Figures 1A, 1B and 1C) contain graphs that summarize the expression level of genes in the C and/or N treatments. The X-axis in these graphs correspond to the treatment carried out as described in the manuscrpt and summarized in the table below . The Y-axis represents the average expression of all genes (Log2 scale) with the indicated regulatory pattern (e.g. +N-independent). The number “n” of genes classified in each pattern is indicated in parenthesis. (A) N-independent patterns (B) C-independent patterns (C) CN interaction patterns.
Treatment	 Sucrose (mM) Nitrate (mM)
1		30	0
2		30	0
3		60	0
4		60	0
5		90	0
6		90	0
7		30	5
8		60	10
9		90	15
10		30	1.7
11		60	3.3
12		90	5
13		30	15
14		60	30
15		90	45
16		30	5
17		60	10
18		90	15
19		30	1.7
20		60	3.3
21		90	5
22		30	15
23		60	30
24		90	45
25		0	5
26		0	5
27		0	10
28		0	10
29		0	15
30		0	15

## Slide 2
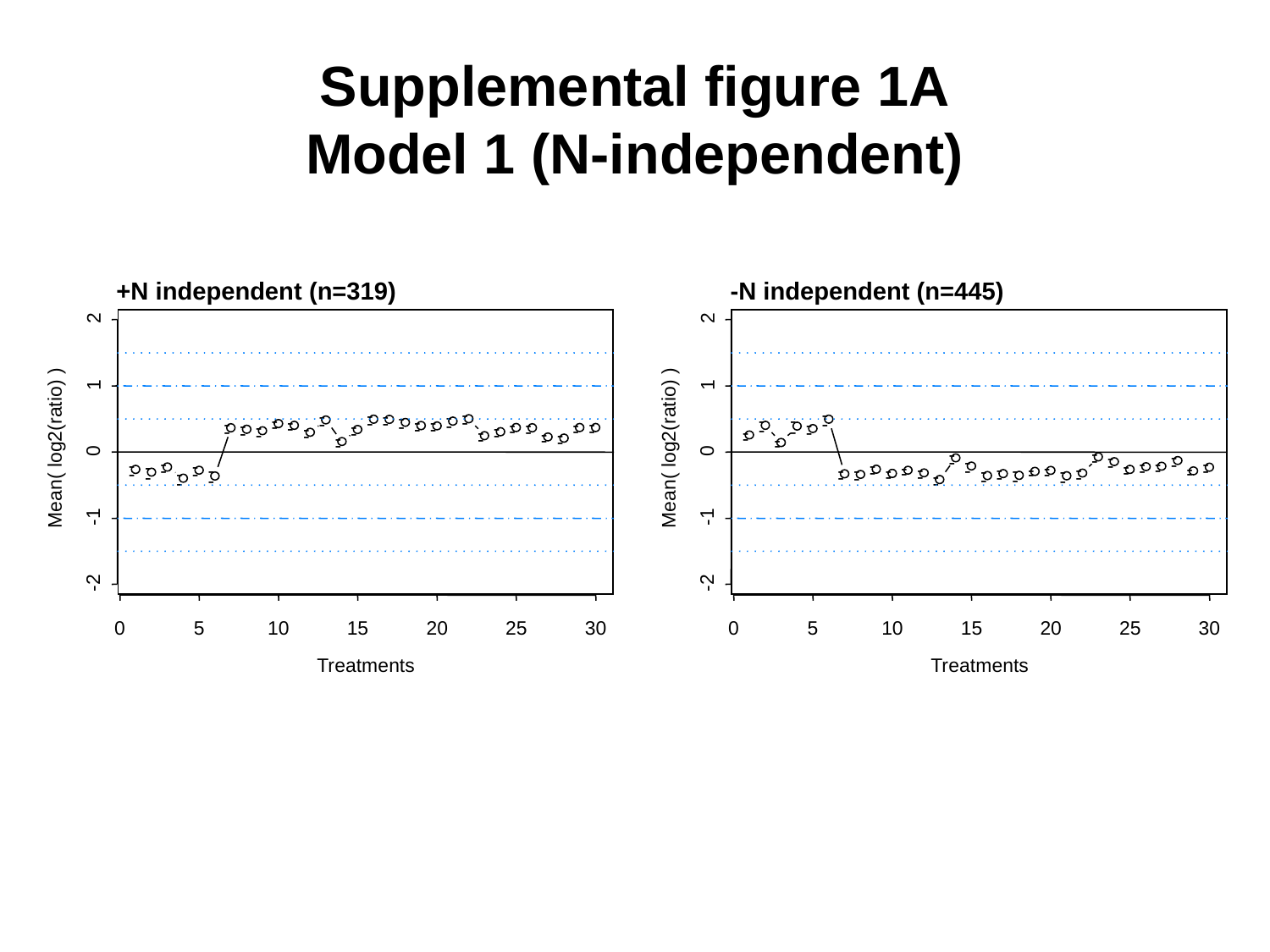

# Supplemental figure 1A Model 1 (N-independent)
+N independent (n=319)
2
1
-
-
-
-
-
-
-
-
-
-
-
-
-
-
-
-
-
-
-
-
-
-
-
-
-
-
-
-
-
-
-
-
-
-
-
-
-
-
-
-
-
-
-
-
-
-
-
-
Mean( log2(ratio) )
0
-
-
-
-
-
-
-
-
-
-
-
-
-1
-2
0
5
10
15
20
25
30
Treatments
-N independent (n=445)
2
1
-
-
-
-
-
-
-
-
-
-
-
-
Mean( log2(ratio) )
0
-
-
-
-
-
-
-
-
-
-
-
-
-
-
-
-
-
-
-
-
-
-
-
-
-
-
-
-
-
-
-
-
-
-
-
-
-
-
-
-
-
-
-
-
-
-
-
-
-1
-2
0
5
10
15
20
25
30
Treatments

## Slide 3
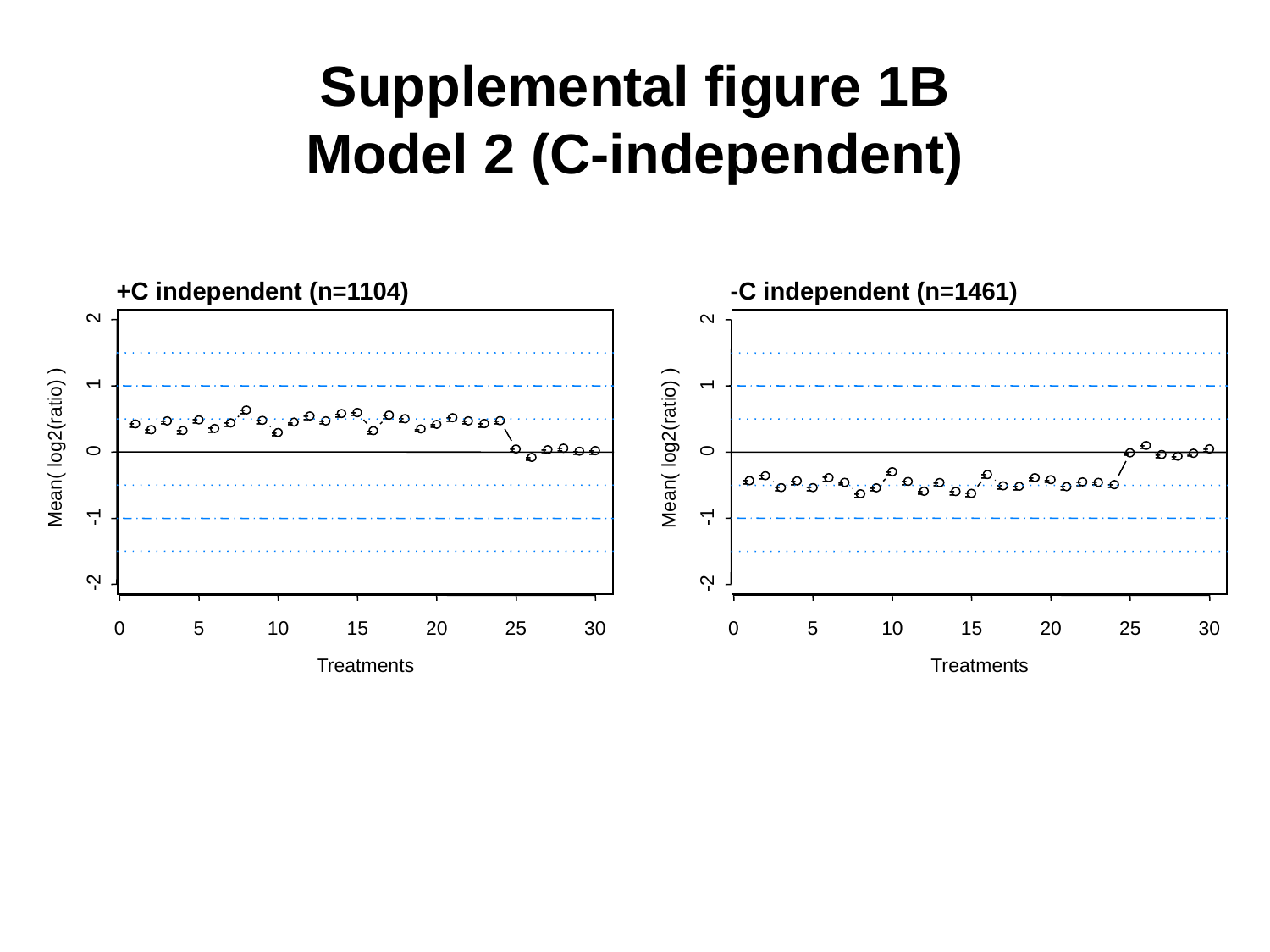

# Supplemental figure 1B Model 2 (C-independent)
+C independent (n=1104)
2
1
-
-
-
-
-
-
-
-
-
-
-
-
-
-
-
-
-
-
-
-
-
-
-
-
-
-
-
-
-
-
-
-
-
-
-
-
-
-
-
-
-
-
-
-
-
-
-
-
-
-
Mean( log2(ratio) )
-
-
-
-
-
-
0
-
-
-
-
-1
-2
0
5
10
15
20
25
30
Treatments
-C independent (n=1461)
2
1
-
-
-
Mean( log2(ratio) )
-
0
-
-
-
-
-
-
-
-
-
-
-
-
-
-
-
-
-
-
-
-
-
-
-
-
-
-
-
-
-
-
-
-
-
-
-
-
-
-
-
-
-
-
-
-
-
-
-
-
-
-
-
-
-
-
-
-
-1
-2
0
5
10
15
20
25
30
Treatments

## Slide 4
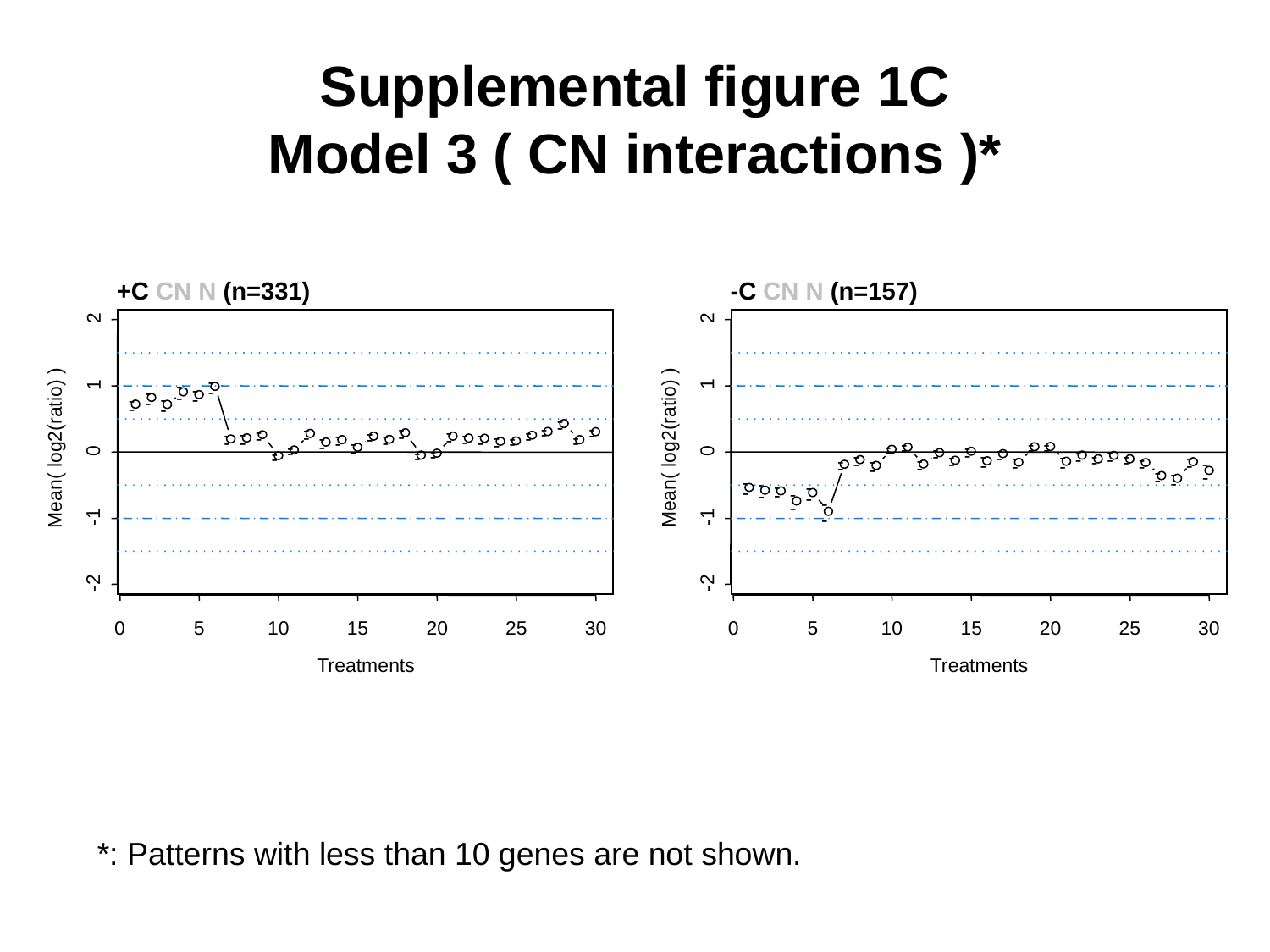

# Supplemental figure 1C Model 3 ( CN interactions )*
+C CN N (n=331)
2
-
1
-
-
-
-
-
-
-
-
-
-
-
-
-
-
-
-
-
-
-
-
-
-
-
-
-
-
-
-
-
-
-
-
-
-
-
-
-
-
-
-
-
-
-
-
-
-
-
-
-
-
-
Mean( log2(ratio) )
-
-
0
-
-
-
-
-
-
-1
-2
0
5
10
15
20
25
30
Treatments
-C CN N (n=157)
2
1
-
-
-
-
-
Mean( log2(ratio) )
-
-
-
-
-
-
0
-
-
-
-
-
-
-
-
-
-
-
-
-
-
-
-
-
-
-
-
-
-
-
-
-
-
-
-
-
-
-
-
-
-
-
-
-
-
-
-
-
-
-
-
-
-
-
-
-1
-
-2
0
5
10
15
20
25
30
Treatments
*: Patterns with less than 10 genes are not shown.

## Slide 5
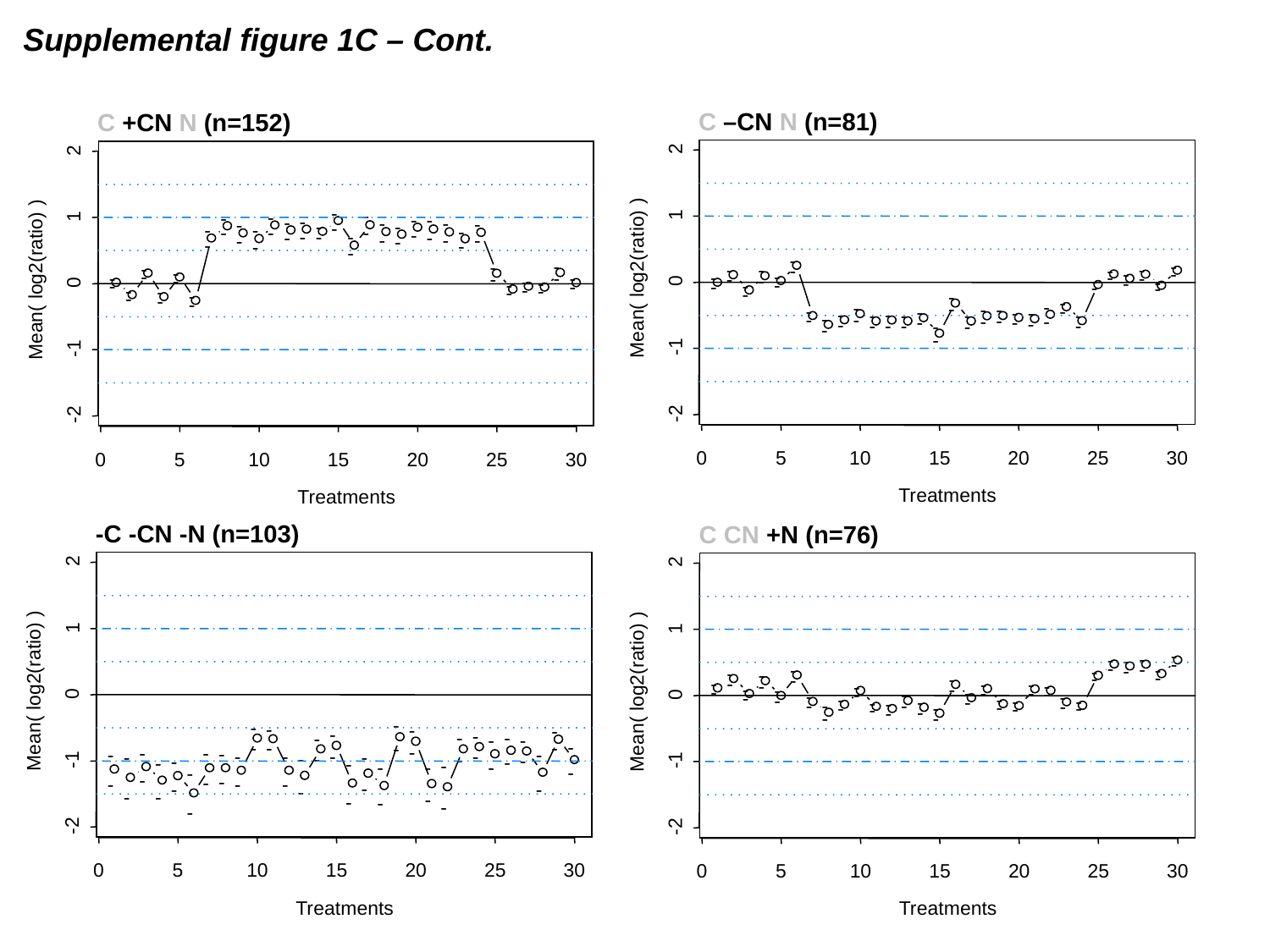

Supplemental figure 1C – Cont.
C –CN N (n=81)
2
1
-
-
-
-
-
-
-
-
-
-
-
-
-
Mean( log2(ratio) )
-
-
-
0
-
-
-
-
-
-
-
-
-
-
-
-
-
-
-
-
-
-
-
-
-
-
-
-
-
-
-
-
-
-
-
-
-
-
-
-
-
-
-
-
-
-
-
-
-1
-2
0
5
10
15
20
25
30
Treatments
C +CN N (n=152)
2
-
-
1
-
-
-
-
-
-
-
-
-
-
-
-
-
-
-
-
-
-
-
-
-
-
-
-
-
-
-
-
-
-
-
-
-
-
-
-
-
-
-
-
-
-
-
Mean( log2(ratio) )
-
-
-
0
-
-
-
-
-
-
-
-
-
-
-
-
-1
-2
0
5
10
15
20
25
30
Treatments
-C -CN -N (n=103)
2
1
Mean( log2(ratio) )
0
-
-
-
-
-
-
-
-
-
-
-
-
-
-
-
-
-
-
-
-
-
-
-
-
-
-
-
-
-
-
-
-
-1
-
-
-
-
-
-
-
-
-
-
-
-
-
-
-
-
-
-
-
-
-
-
-
-
-
-
-
-
-2
0
5
10
15
20
25
30
Treatments
C CN +N (n=76)
2
1
-
-
-
-
-
-
-
-
-
-
-
-
-
-
-
-
-
-
-
-
-
-
-
-
-
-
-
-
Mean( log2(ratio) )
-
-
-
-
-
-
0
-
-
-
-
-
-
-
-
-
-
-
-
-
-
-
-
-
-
-
-
-
-
-
-
-
-
-1
-2
0
5
10
15
20
25
30
Treatments

## Slide 6
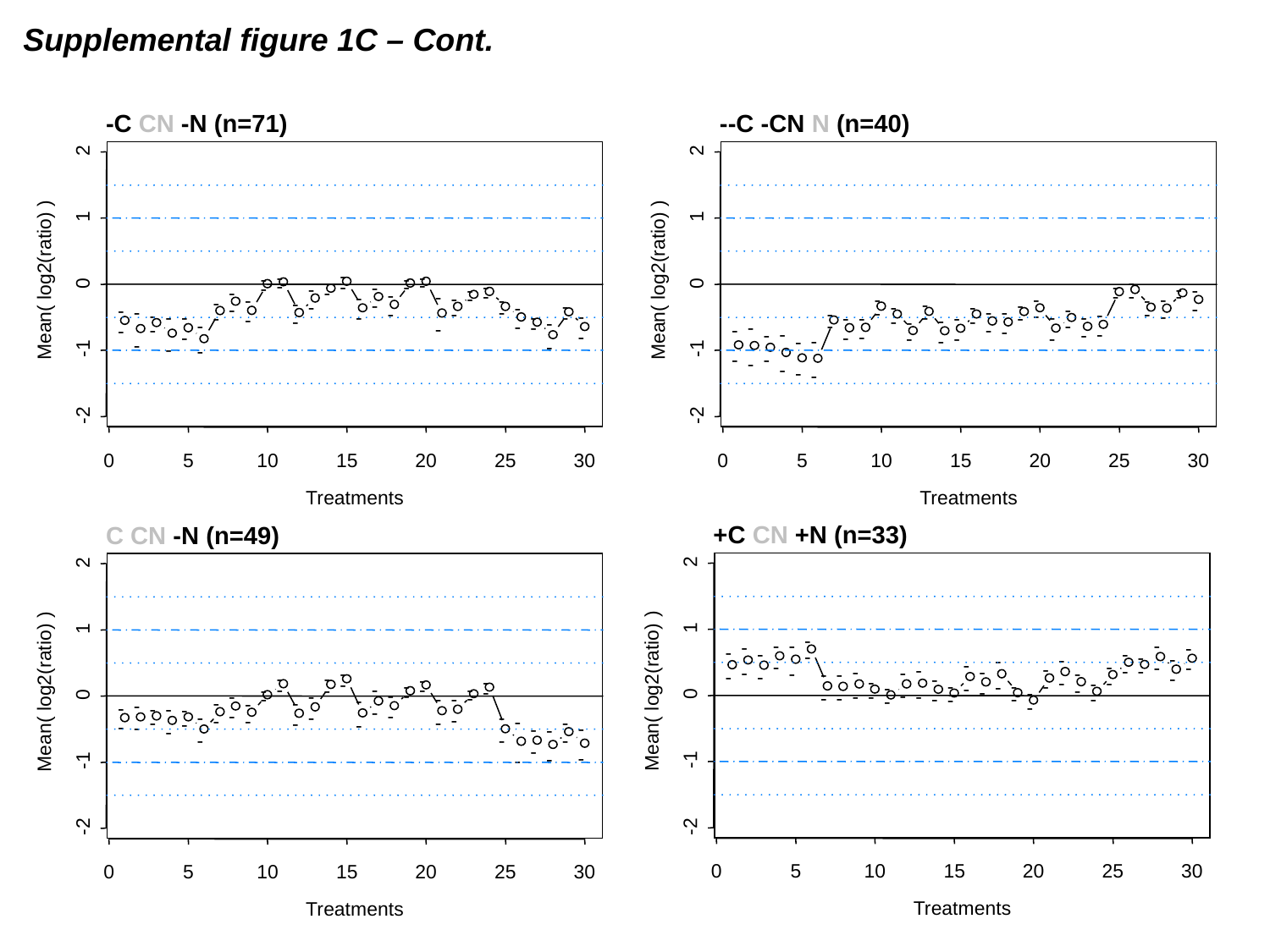

Supplemental figure 1C – Cont.
-C CN -N (n=71)
2
1
-
-
-
-
-
Mean( log2(ratio) )
-
0
-
-
-
-
-
-
-
-
-
-
-
-
-
-
-
-
-
-
-
-
-
-
-
-
-
-
-
-
-
-
-
-
-
-
-
-
-
-
-
-
-
-
-
-
-
-
-
-
-
-
-
-
-
-1
-
-2
0
5
10
15
20
25
30
Treatments
--C -CN N (n=40)
2
1
Mean( log2(ratio) )
-
0
-
-
-
-
-
-
-
-
-
-
-
-
-
-
-
-
-
-
-
-
-
-
-
-
-
-
-
-
-
-
-
-
-
-
-
-
-
-
-
-
-
-
-
-
-
-
-
-
-
-
-
-
-
-1
-
-
-
-
-
-
-2
0
5
10
15
20
25
30
Treatments
+C CN +N (n=33)
2
1
-
-
-
-
-
-
-
-
-
-
-
-
-
-
-
-
-
-
-
-
-
-
-
-
-
-
-
-
-
-
-
-
-
-
-
-
-
-
-
-
-
-
-
-
-
-
Mean( log2(ratio) )
-
-
0
-
-
-
-
-
-
-
-
-
-
-
-
-1
-2
0
5
10
15
20
25
30
Treatments
C CN -N (n=49)
2
1
-
-
-
-
-
-
-
-
-
-
-
-
-
Mean( log2(ratio) )
-
-
-
0
-
-
-
-
-
-
-
-
-
-
-
-
-
-
-
-
-
-
-
-
-
-
-
-
-
-
-
-
-
-
-
-
-
-
-
-
-
-
-
-
-
-
-
-
-1
-2
0
5
10
15
20
25
30
Treatments

## Slide 7
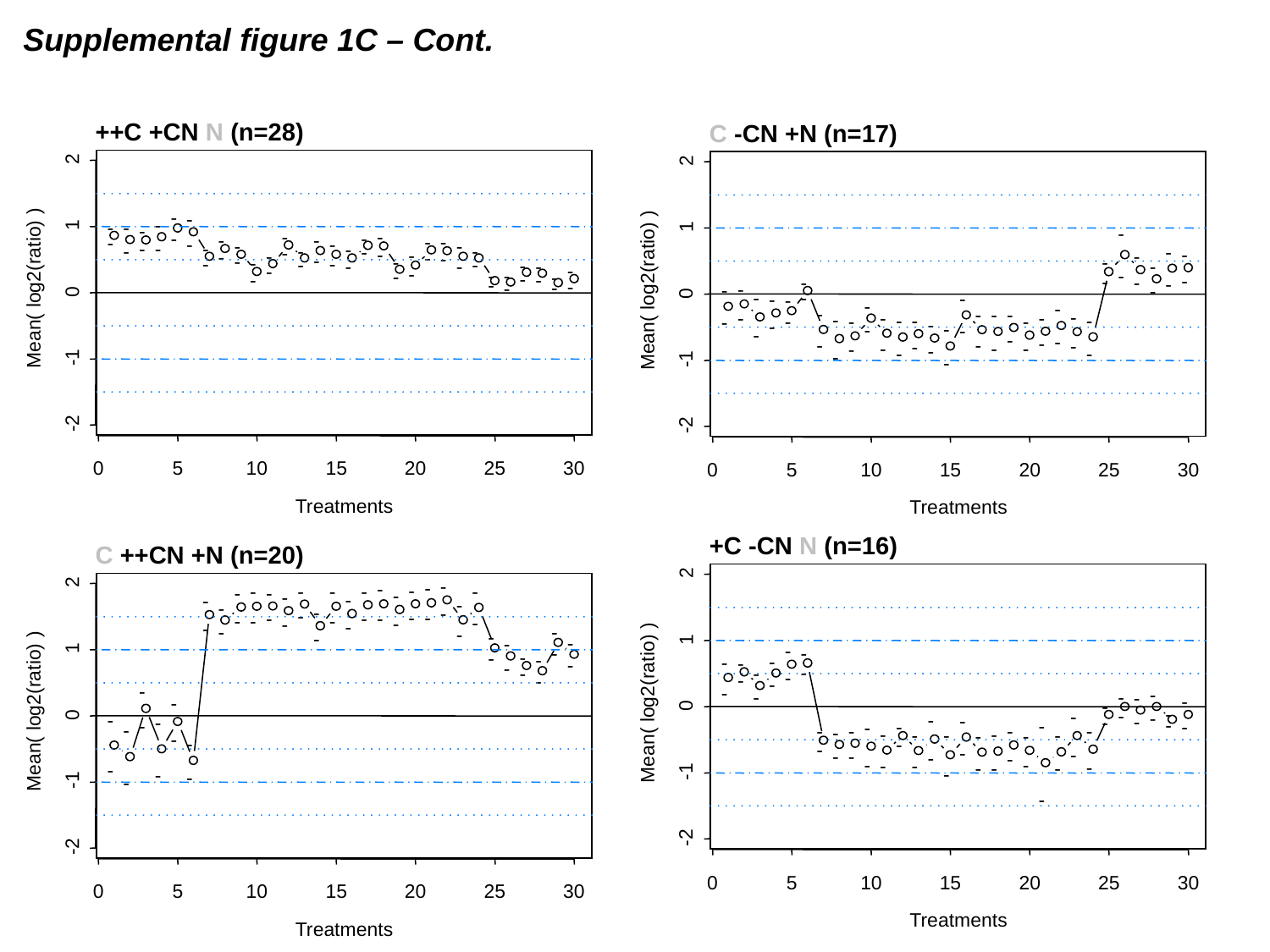

Supplemental figure 1C – Cont.
++C +CN N (n=28)
2
-
-
-
1
-
-
-
-
-
-
-
-
-
-
-
-
-
-
-
-
-
-
-
-
-
-
-
-
-
-
-
-
-
-
-
-
-
-
-
-
-
-
-
-
-
-
-
-
-
-
-
-
-
-
-
-
-
-
-
-
-
Mean( log2(ratio) )
0
-1
-2
0
5
10
15
20
25
30
Treatments
C -CN +N (n=17)
2
1
-
-
-
-
-
-
-
-
-
-
-
-
-
-
Mean( log2(ratio) )
-
0
-
-
-
-
-
-
-
-
-
-
-
-
-
-
-
-
-
-
-
-
-
-
-
-
-
-
-
-
-
-
-
-
-
-
-
-
-
-
-
-
-
-
-
-
-1
-
-2
0
5
10
15
20
25
30
Treatments
+C -CN N (n=16)
2
1
-
-
-
-
-
-
-
-
-
-
-
-
-
-
-
-
Mean( log2(ratio) )
0
-
-
-
-
-
-
-
-
-
-
-
-
-
-
-
-
-
-
-
-
-
-
-
-
-
-
-
-
-
-
-
-
-
-
-
-
-
-
-
-
-
-
-1
-
-
-2
0
5
10
15
20
25
30
Treatments
C ++CN +N (n=20)
2
-
-
-
-
-
-
-
-
-
-
-
-
-
-
-
-
-
-
-
-
-
-
-
-
-
-
-
-
-
-
-
-
-
-
-
-
-
-
-
-
1
-
-
-
-
-
-
-
-
-
-
Mean( log2(ratio) )
0
-
-
-
-
-
-
-
-
-
-1
-
-2
0
5
10
15
20
25
30
Treatments

## Slide 8
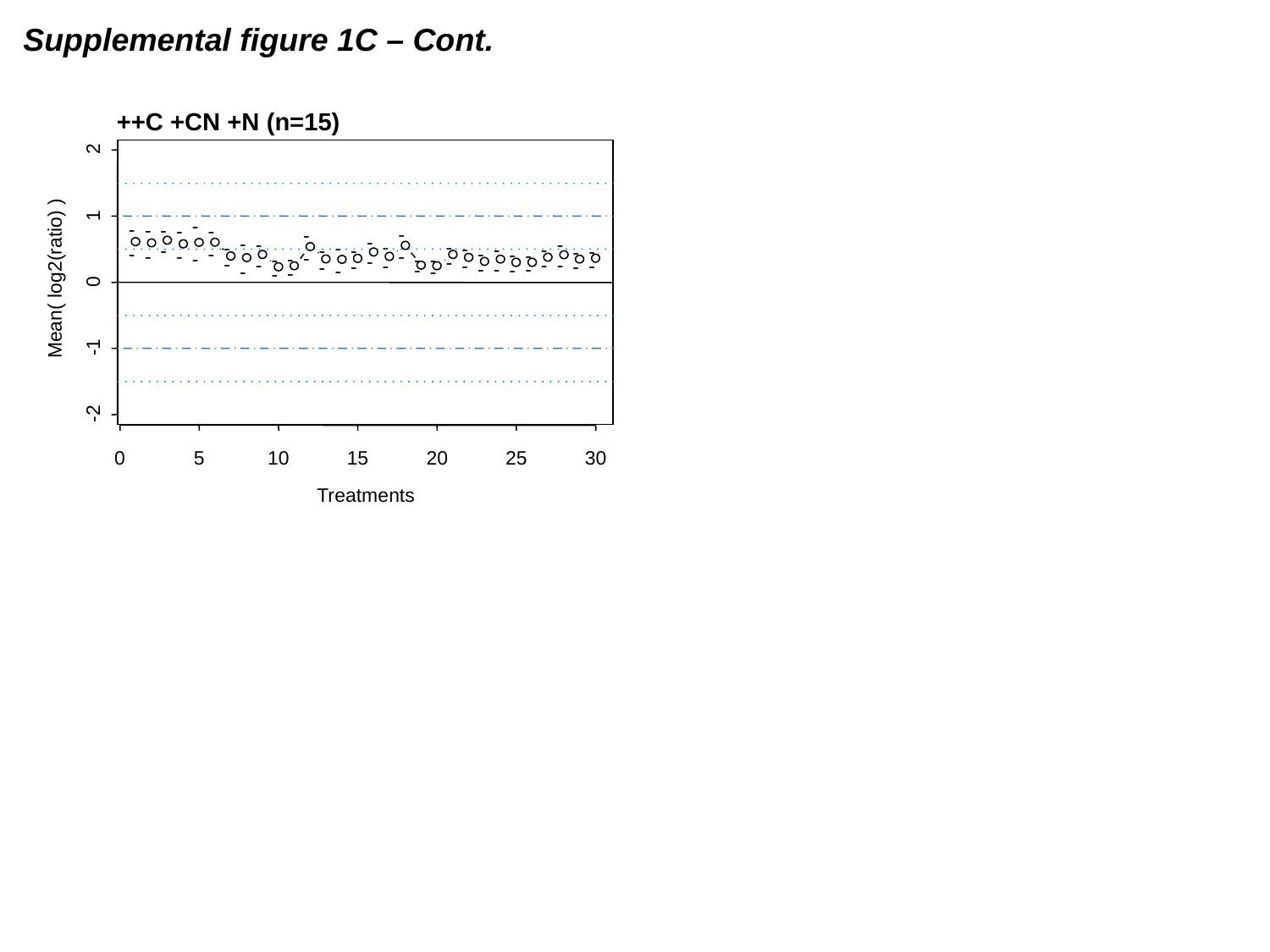

Supplemental figure 1C – Cont.
++C +CN +N (n=15)
2
1
-
-
-
-
-
-
-
-
-
-
-
-
-
-
-
-
-
-
-
-
-
-
-
-
-
-
-
-
-
-
-
-
-
-
-
-
-
-
-
-
-
-
-
-
-
-
-
-
-
-
-
-
-
-
-
-
-
-
-
-
Mean( log2(ratio) )
0
-1
-2
0
5
10
15
20
25
30
Treatments

## Slide 9
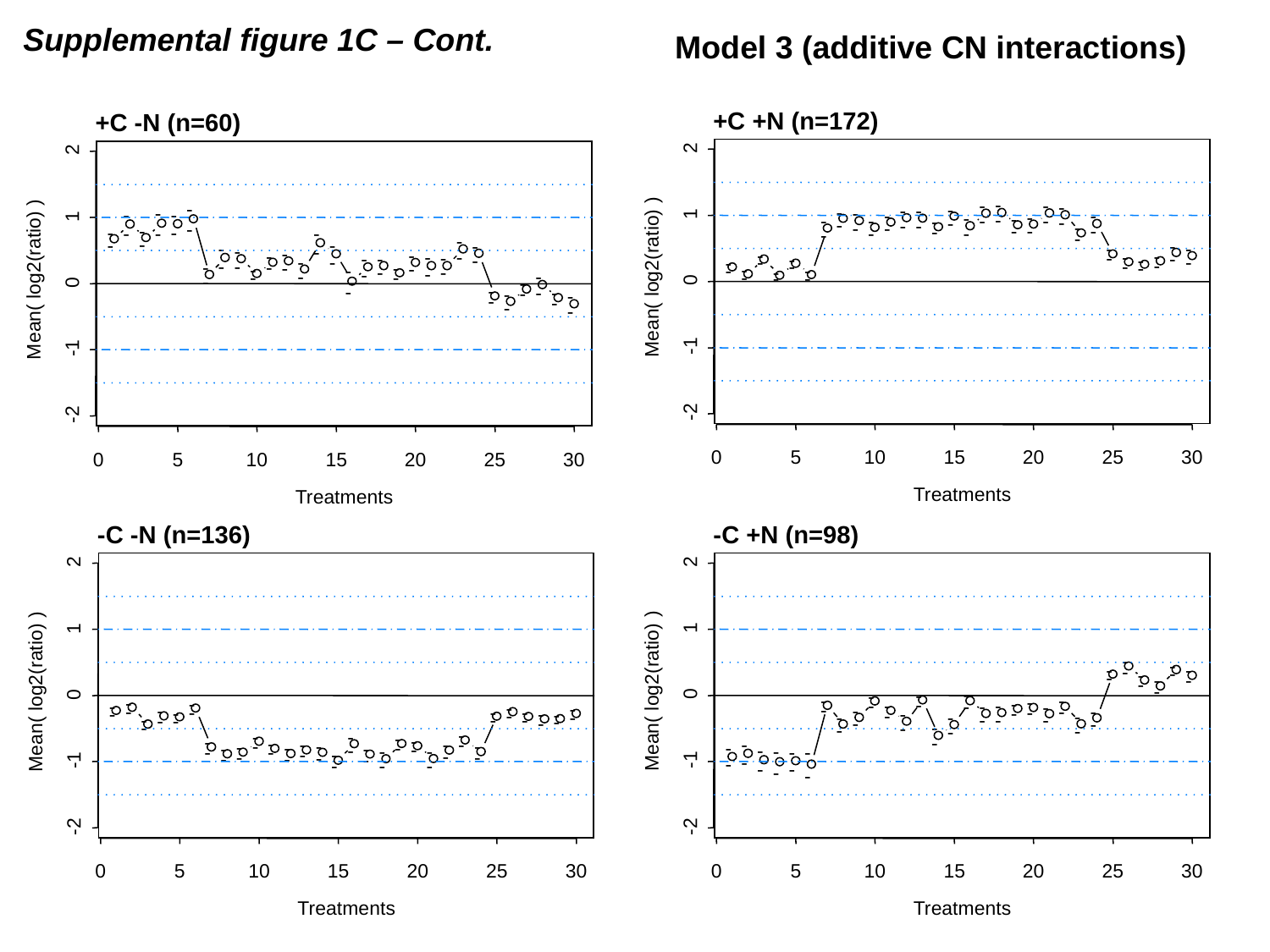

Supplemental figure 1C – Cont.
Model 3 (additive CN interactions)
+C +N (n=172)
+C -N (n=60)
2
-
-
-
-
1
-
-
-
-
-
-
-
-
-
-
-
-
-
-
-
-
-
-
-
-
-
-
-
-
-
-
-
-
-
-
-
-
-
-
-
-
-
-
-
-
-
-
-
Mean( log2(ratio) )
-
0
-
-
-
-
-
-
-
-
-
-
-
-
-1
-2
0
5
10
15
20
25
30
Treatments
2
-
-
-
-
-
-
-
-
-
1
-
-
-
-
-
-
-
-
-
-
-
-
-
-
-
-
-
-
-
-
-
-
-
-
-
-
-
-
-
-
-
-
-
-
-
-
-
-
-
-
-
-
-
-
-
-
-
-
-
Mean( log2(ratio) )
-
-
0
-1
-2
0
5
10
15
20
25
30
Treatments
-C -N (n=136)
-C +N (n=98)
2
2
1
1
-
-
-
-
-
-
-
-
-
-
-
-
Mean( log2(ratio) )
Mean( log2(ratio) )
0
-
-
0
-
-
-
-
-
-
-
-
-
-
-
-
-
-
-
-
-
-
-
-
-
-
-
-
-
-
-
-
-
-
-
-
-
-
-
-
-
-
-
-
-
-
-
-
-
-
-
-
-
-
-
-
-
-
-
-
-
-
-
-
-
-
-
-
-
-
-
-
-
-
-
-
-
-
-
-
-
-
-
-
-
-
-
-
-
-
-
-
-
-
-
-
-
-
-
-
-
-1
-1
-
-
-
-
-
-
-
-
-
-2
-2
0
5
10
15
20
25
30
0
5
10
15
20
25
30
Treatments
Treatments

## Slide 10
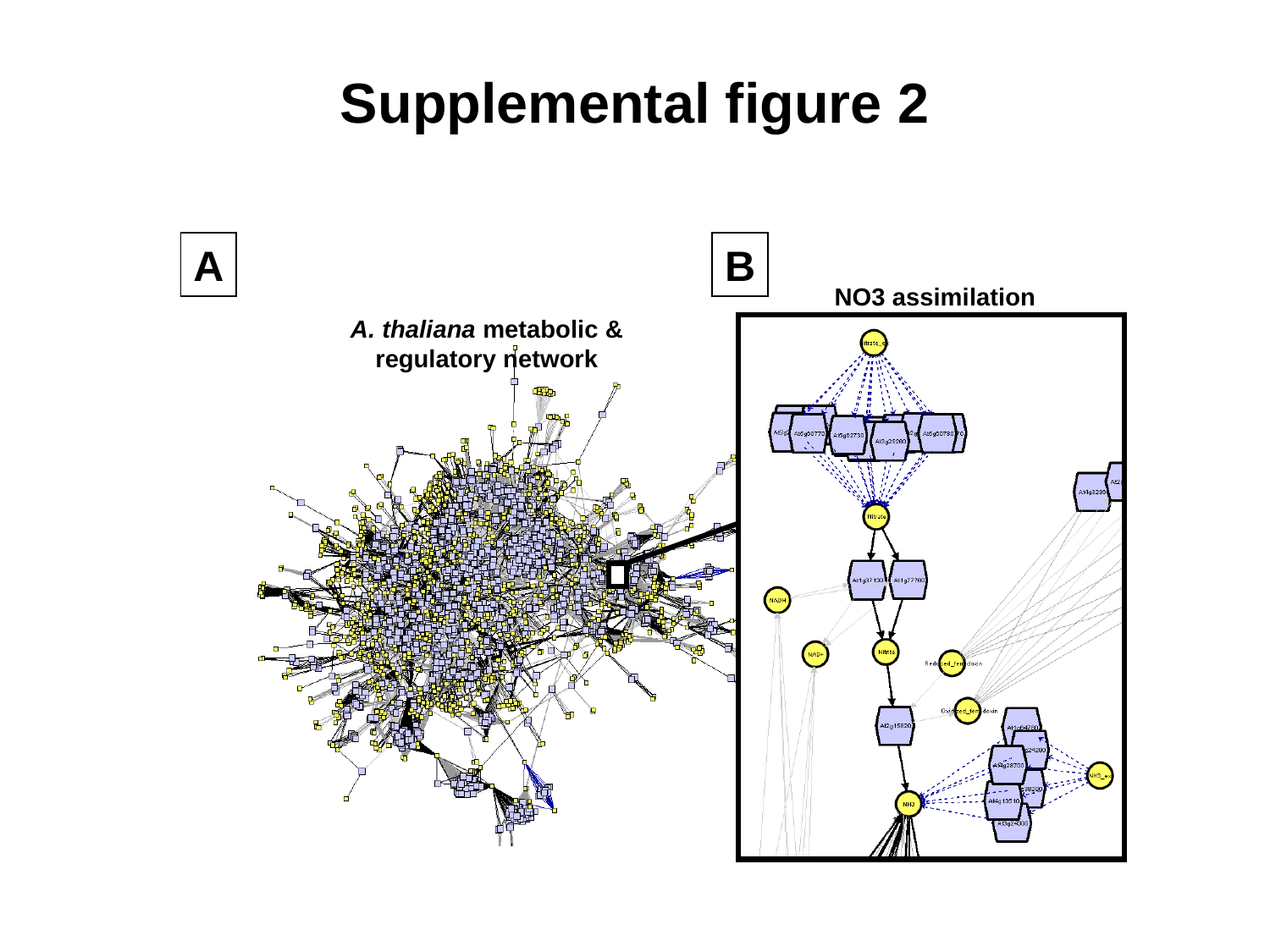

# Supplemental figure 2
A
B
NO3 assimilation
A. thaliana metabolic & regulatory network

## Slide 11
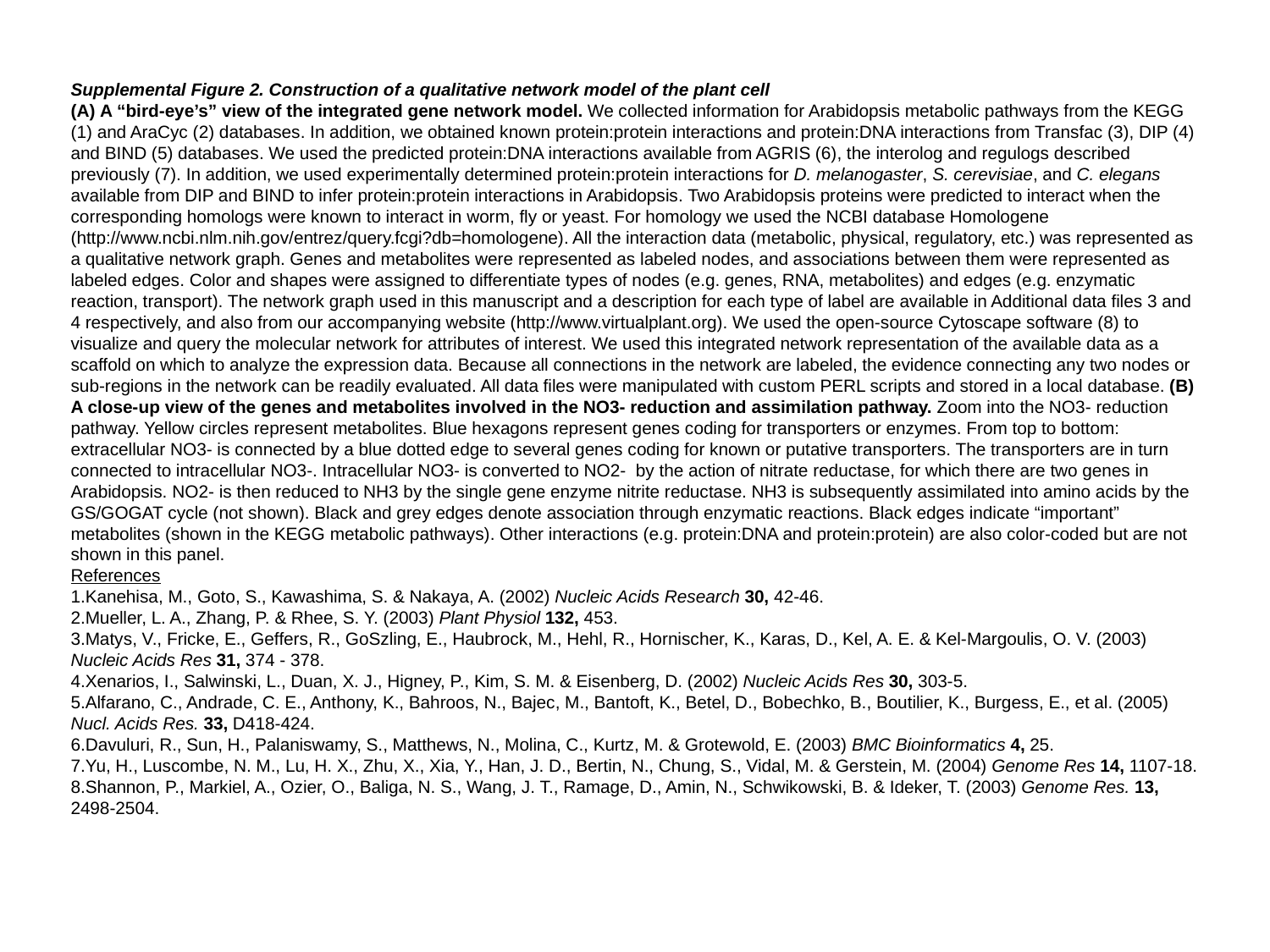

Supplemental Figure 2. Construction of a qualitative network model of the plant cell
(A) A “bird-eye’s” view of the integrated gene network model. We collected information for Arabidopsis metabolic pathways from the KEGG (1) and AraCyc (2) databases. In addition, we obtained known protein:protein interactions and protein:DNA interactions from Transfac (3), DIP (4) and BIND (5) databases. We used the predicted protein:DNA interactions available from AGRIS (6), the interolog and regulogs described previously (7). In addition, we used experimentally determined protein:protein interactions for D. melanogaster, S. cerevisiae, and C. elegans available from DIP and BIND to infer protein:protein interactions in Arabidopsis. Two Arabidopsis proteins were predicted to interact when the corresponding homologs were known to interact in worm, fly or yeast. For homology we used the NCBI database Homologene (http://www.ncbi.nlm.nih.gov/entrez/query.fcgi?db=homologene). All the interaction data (metabolic, physical, regulatory, etc.) was represented as a qualitative network graph. Genes and metabolites were represented as labeled nodes, and associations between them were represented as labeled edges. Color and shapes were assigned to differentiate types of nodes (e.g. genes, RNA, metabolites) and edges (e.g. enzymatic reaction, transport). The network graph used in this manuscript and a description for each type of label are available in Additional data files 3 and 4 respectively, and also from our accompanying website (http://www.virtualplant.org). We used the open-source Cytoscape software (8) to visualize and query the molecular network for attributes of interest. We used this integrated network representation of the available data as a scaffold on which to analyze the expression data. Because all connections in the network are labeled, the evidence connecting any two nodes or sub-regions in the network can be readily evaluated. All data files were manipulated with custom PERL scripts and stored in a local database. (B) A close-up view of the genes and metabolites involved in the NO3- reduction and assimilation pathway. Zoom into the NO3- reduction pathway. Yellow circles represent metabolites. Blue hexagons represent genes coding for transporters or enzymes. From top to bottom: extracellular NO3- is connected by a blue dotted edge to several genes coding for known or putative transporters. The transporters are in turn connected to intracellular NO3-. Intracellular NO3- is converted to NO2- by the action of nitrate reductase, for which there are two genes in Arabidopsis. NO2- is then reduced to NH3 by the single gene enzyme nitrite reductase. NH3 is subsequently assimilated into amino acids by the GS/GOGAT cycle (not shown). Black and grey edges denote association through enzymatic reactions. Black edges indicate “important” metabolites (shown in the KEGG metabolic pathways). Other interactions (e.g. protein:DNA and protein:protein) are also color-coded but are not shown in this panel.
References
1.Kanehisa, M., Goto, S., Kawashima, S. & Nakaya, A. (2002) Nucleic Acids Research 30, 42-46.
2.Mueller, L. A., Zhang, P. & Rhee, S. Y. (2003) Plant Physiol 132, 453.
3.Matys, V., Fricke, E., Geffers, R., GoSzling, E., Haubrock, M., Hehl, R., Hornischer, K., Karas, D., Kel, A. E. & Kel-Margoulis, O. V. (2003) Nucleic Acids Res 31, 374 - 378.
4.Xenarios, I., Salwinski, L., Duan, X. J., Higney, P., Kim, S. M. & Eisenberg, D. (2002) Nucleic Acids Res 30, 303-5.
5.Alfarano, C., Andrade, C. E., Anthony, K., Bahroos, N., Bajec, M., Bantoft, K., Betel, D., Bobechko, B., Boutilier, K., Burgess, E., et al. (2005) Nucl. Acids Res. 33, D418-424.
6.Davuluri, R., Sun, H., Palaniswamy, S., Matthews, N., Molina, C., Kurtz, M. & Grotewold, E. (2003) BMC Bioinformatics 4, 25.
7.Yu, H., Luscombe, N. M., Lu, H. X., Zhu, X., Xia, Y., Han, J. D., Bertin, N., Chung, S., Vidal, M. & Gerstein, M. (2004) Genome Res 14, 1107-18.
8.Shannon, P., Markiel, A., Ozier, O., Baliga, N. S., Wang, J. T., Ramage, D., Amin, N., Schwikowski, B. & Ideker, T. (2003) Genome Res. 13, 2498-2504.

## Slide 12
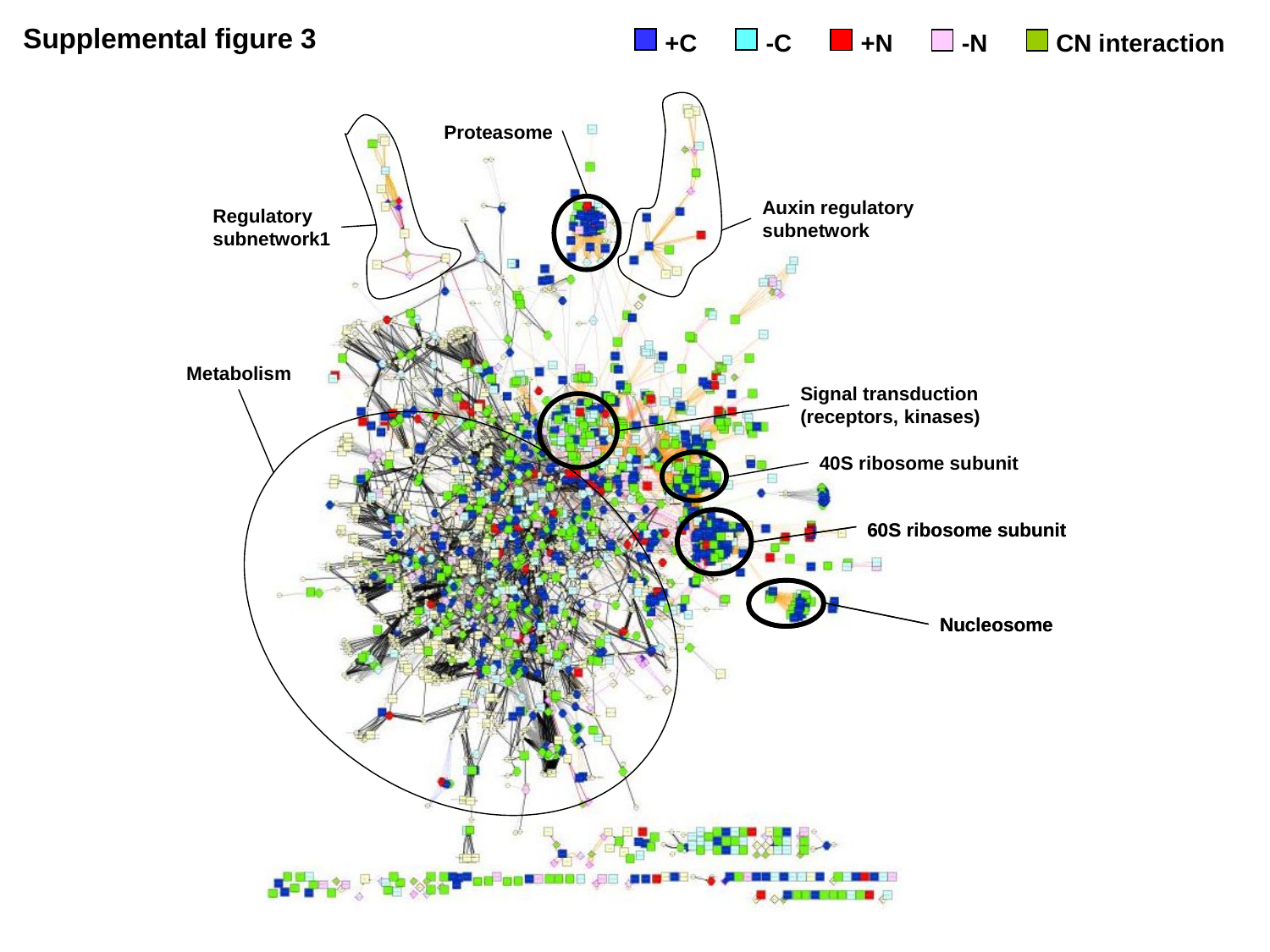

Supplemental figure 3
+C
-C
+N
-N
CN interaction

## Slide 13
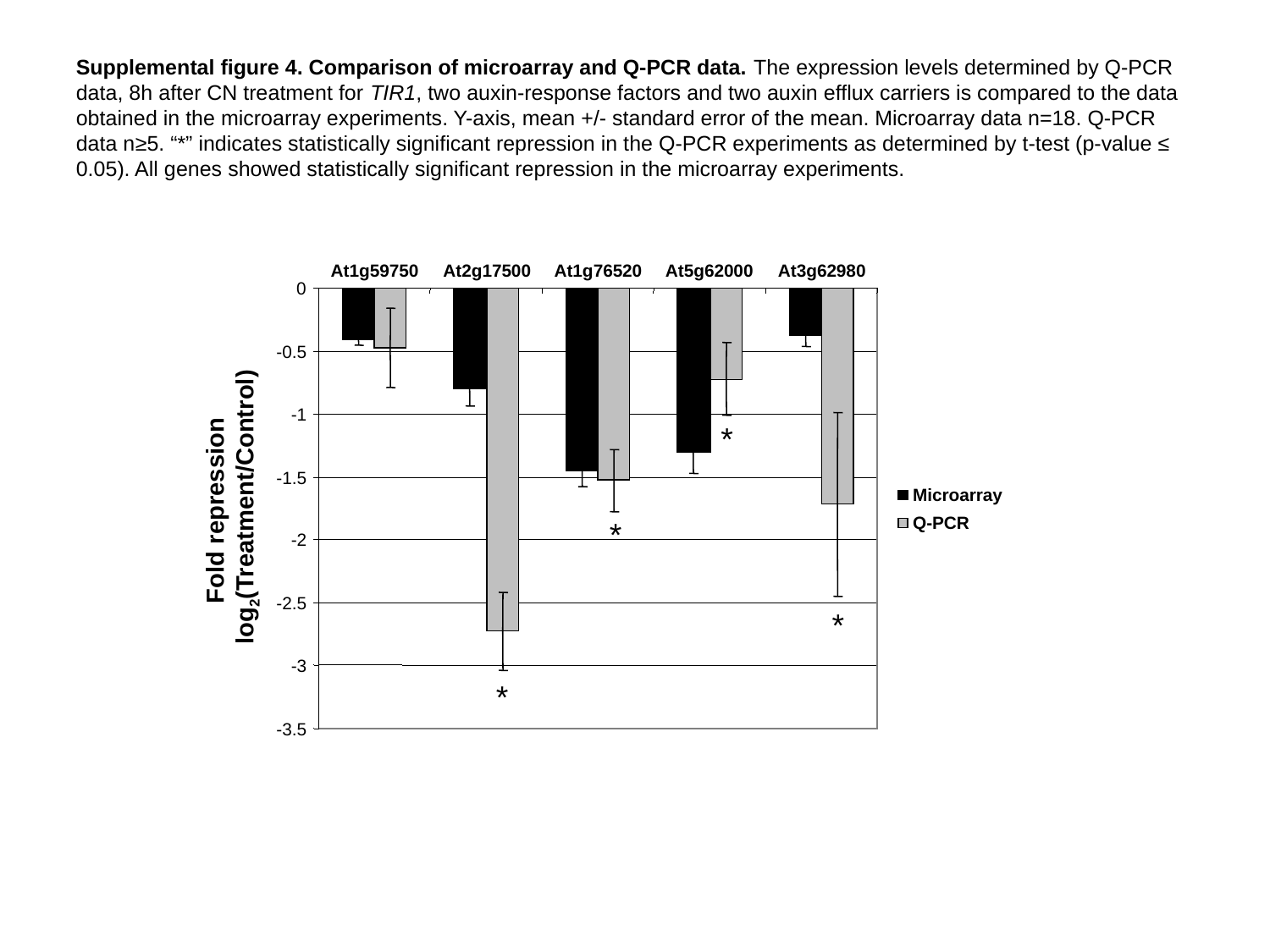

# Supplemental figure 4. Comparison of microarray and Q-PCR data. The expression levels determined by Q-PCR data, 8h after CN treatment for TIR1, two auxin-response factors and two auxin efflux carriers is compared to the data obtained in the microarray experiments. Y-axis, mean +/- standard error of the mean. Microarray data n=18. Q-PCR data n≥5. “*” indicates statistically significant repression in the Q-PCR experiments as determined by t-test (p-value ≤ 0.05). All genes showed statistically significant repression in the microarray experiments.
At1g59750
At2g17500
At1g76520
At5g62000
At3g62980
0
-0.5
-1
*
-1.5
Fold repression
log2(Treatment/Control)
Microarray
*
Q-PCR
-2
-2.5
*
-3
*
-3.5
